# *GBA* and *APOE ε4* associate with sporadic dementia with Lewy bodies in European genome wide association study

**DOI:** 10.1038/s41598-019-43458-2

**Published:** 2019-05-07

**Authors:** Arvid Rongve, Aree Witoelar, Agustín Ruiz, Lavinia Athanasiu, Carla Abdelnour, Jordi Clarimon, Stefanie Heilmann-Heimbach, Isabel Hernández, Sonia Moreno-Grau, Itziar de Rojas, Estrella Morenas-Rodríguez, Tormod Fladby, Sigrid B. Sando, Geir Bråthen, Frédéric Blanc, Olivier Bousiges, Afina W. Lemstra, Inger van Steenoven, Elisabet Londos, Ina S. Almdahl, Lene Pålhaugen, Jon A. Eriksen, Srdjan Djurovic, Eystein Stordal, Ingvild Saltvedt, Ingun D. Ulstein, Francesco Bettella, Rahul S. Desikan, Ane-Victoria Idland, Mathias Toft, Lasse Pihlstrøm, Jon Snaedal, Lluís Tárraga, Mercè Boada, Alberto Lleó, Hreinn Stefánsson, Kári Stefánsson, Alfredo Ramírez, Dag Aarsland, Ole A. Andreassen

**Affiliations:** 1 Haugesund Hospital, Helse Fonna, Department of Research and Innovation, Haugesund, Norway; 2 The University of Bergen, Department of Clinical Medicine (K1), Bergen, Norway; 3 NORMENT, KG Jebsen Centre for Psychosis Research, Division of Mental Health and Addiction, Oslo University Hospital, Oslo, Norway; 4 Institute of Clinical Medicine, University of Oslo, Oslo, Norway; 5 Memory Clinic and Research Center of Fundació ACE, Institut Català de Neurociències Aplicades, Universitat Internacional de Catalunya (UIC), Barcelona, Spain; 6 Department of Neurology, IIB Sant Pau, Hospital de la Santa Creu i Sant Pau, Universitat Autònoma de Barcelona, Barcelona, Spain; 7 Center for Networker Biomedical Research in Neurodegenerative Diseases (CIBERNED), Madrid and Barcelona, Spain; 8 Institute of Human Genetics, University of Bonn, Bonn, Germany; 9 Department of Genomics, Life & Brain Center, University of Bonn, Bonn, Germany; 10 Department of Neurology, Akershus University Hospital, Lørenskog, Norway; 11 University of Oslo, AHUS Campus, Oslo, Norway; 12 Department of Neuromedicine and Movement Science, Norwegian University of Science and Technology, Trondheim, Norway; 13 Department of Neurology, St Olav’s Hospital, Trondheim, Norway; 14 University Hospital of Strasbourg, CMRR (Memory Resources and Research Centre), Geriatrics Department, Strasbourg, France; 15 University of Strasbourg and CNRS, ICube laboratory and FMTS, team IMIS/Neurocrypto, Strasbourg, France; 16 University Hospital of Strasbourg, CMRR (Memory Resources and Research Centre), Laboratory of Biochemistry and Molecular Biology, Strasbourg, France; 17 University of Strasbourg and CNRS, Laboratoire de Neurosciences Cognitives et Adaptatives (LNCA), UMR7364, 67000 Strasbourg, France; 18 Alzheimercenter & Department of Neurology VU University Medical Center, Amsterdam, the Netherlands; 19 Lund University, Skane University Hospital, Institute of Clinical Sciences, Malmö, Sweden; 20 Department of Medical Genetics, Oslo University Hospital, Oslo, Norway; 21 NORMENT, KG Jebsen Centre for Psychosis Research, Department of Clinical Science, University of Bergen, Bergen, Norway; 22 Department of Psychiatry, Namsos Hospital, Namsos, Norway; 23 Department of Mental Health, Norwegian University of Science and Technology, Trondheim, Norway; 24 Department of Geriatrics, St. Olav’s Hospital, Trondheim, Norway; 25 Department of Geriatric Psychiatry, Oslo University Hospital, Oslo, Norway; 26 Departments of Radiology and Biomedical Imaging, Neurology and Pediatrics, UCSF, San Francisco, USA; 27 Oslo Delirium Research Group, Department of Geriatric Medicine, Institute of Clinical Medicine, University of Oslo, Oslo, Norway; 28 Research Group for Lifespan Changes in Brain and Cognition, Department of Psychology, University of Oslo, Oslo, Norway; 29 Institute of Basic Medical Sciences, University of Oslo, Oslo, Norway; 30 Department of Neurology, Oslo University Hospital, Oslo, Norway; 31 Landspitali University Hospital, Reykjavik, Iceland; 32 DeCODE genetics, Reykjavik, Iceland; 33 Division for Neurogenetics and Molecular Psychiatry, Department of Psychiatry and Psychotherapy, Medical Faculty, University of Cologne, 50924 Cologne, Germany; 34 Department for Neurodegenerative Diseases and Geriatric Psychiatry, University of Bonn, 53127 Bonn, Germany; 35 Institute of Psychiatry, Psychology and Neuroscience, King’s College London, London, UK; 36 Center for Age-Related Diseases, Stavanger University Hospital, Stavanger, Norway

**Keywords:** Molecular medicine, Alzheimer's disease

## Abstract

Dementia with Lewy Bodies (DLB) is a common neurodegenerative disorder with poor prognosis and mainly unknown pathophysiology. Heritability estimates exceed 30% but few genetic risk variants have been identified. Here we investigated common genetic variants associated with DLB in a large European multisite sample. We performed a genome wide association study in Norwegian and European cohorts of 720 DLB cases and 6490 controls and included 19 top-associated single-nucleotide polymorphisms in an additional cohort of 108 DLB cases and 75545 controls from Iceland. Overall the study included 828 DLB cases and 82035 controls. Variants in the *ASH1L/GBA* (Chr1q22) and *APOE ε4* (Chr19) loci were associated with DLB surpassing the genome-wide significance threshold (p < 5 × 10^−8^). One additional genetic locus previously linked to psychosis in Alzheimer’s disease, *ZFPM1* (Chr16q24.2), showed suggestive association with DLB at p-value < 1 × 10^−6^. We report two susceptibility loci for DLB at genome-wide significance, providing insight into etiological factors. These findings highlight the complex relationship between the genetic architecture of DLB and other neurodegenerative disorders.

## Introduction

Dementia with Lewy Bodies (DLB) is the second most common type of neurodegenerative dementia, accounting for 15% of dementia patients. DLB overlaps clinically, pathologically and genetically with Alzheimer’s disease (AD) and Parkinson’s disease (PD). Clinically, DLB is characterized by cognitive impairment, parkinsonism, psychotic symptoms like visual hallucinations, fluctuating cognition with pronounced variations in attention and alertness and REM sleep behaviour disorder. Reduced uptake on CIT-SPECT or myocardial scintigraphy and polysomnography with confirmation of REM sleep without atonia have been included as indicative biomarkers in the diagnostic criteria^1^. We have previously shown the clinical diagnostic criteria for probable DLB to be both sensitive (77%) and highly specific (94%) as compared to a pathological DLB diagnosis. Furthermore, we have found DLB to have higher costs, more neuropsychiatric symptoms, a more rapid cognitive decline, shorter time until nursing home admission, shorter survival and higher caregiver distress as compared to AD^2–8^.

In some families, DLB occurs with autosomal dominant heritance at an age of onset <65 years. In these families, alpha-synuclein (*SNCA*) multiplications or point mutations have been described^9,10^. DLB is however typically late onset (i.e. onset after 65 years of age) and sporadic, and the proportion of phenotypic variance that can be explained by >250,000 SNPs on the NeuroX array has been estimated to 31% with substantial genetic overlap with both AD and PD^11^. Indeed, previous genetic studies have suggested associations of *APOE*, *GBA*, *SNCA* and *SCARB2* with DLB in both neuropathologically and clinically diagnosed cases^12^. Data from another GWAS of DLB were recently presented and confirmed *APOE e4*, *SNCA* and *GBA*, and in addition suggested *CNTN1* to be associated with DLB^13^. Regarding *APOE*, the strongest genetic risk factor for AD, we showed that the *APOE ε4* allele increases and the *APOE ε2* allele decreases the risk of developing DLB^14^. *GBA*, the gene encoding the lysosomal enzyme glucocerebrosidase, is associated with PD risk and cognitive decline in PD^15,16^. In DLB, *GBA* mutations have been reported in 7.8% of cases (odds ratio (OR) ~8), even up to 31% in Ashkenazi Jews, suggesting that *GBA* is a stronger risk factor for DLB than for PD^17^. Moreover, whole exome sequencing studies have identified rare and pathogenic variants in *GBA*, *PSEN1* or *APP* in 4.4–25% of patients with pathologically or clinically diagnosed DLB^18,19^. However, none of the AD associated common variants identified in large genome-wide association studies (GWAS) have been associated to DLB besides *APOE*.

Notwithstanding these interesting results from early genetic studies, the individual genetic risk factors that specifically contribute to the common and sporadic late onset form of DLB have remained relatively unexplored compared to PD and AD, largely due to the lack of large sample series providing adequate statistical power for GWAS. In the current study, we collected DNA and genotyped samples from the Norwegian DemGene consortium and the European DLB consortium (E-DLB), and performed a GWAS applying a two-stage meta-analysis approach and follow-up in an independent cohort. We investigated whether common genetic variants are associated with DLB, aiming to elucidate the molecular mechanisms underlying the disease.

## Methods

### Participants

Three discovery cohorts were included in the study (Cohorts 1, 2 and 3). We collected samples from the Norwegian Dementia Genetics Network (DemGene) and from the European DLB consortium (E-DLB). Cohort 1 included DemGene and three European centres (Strasbourg, Amsterdam and Lund), and consisted of 478 cases and 1322 controls. An additional Norwegian population cohort of 4875 controls was added. Cohort 2 included two European centres both from Barcelona and consisted of 242 cases and 293 controls. Cohort 3 samples were collected in Iceland and consisted of 108 cases and 75545 controls. Altogether 828 DLB cases and 82110 controls were included in this study, see Supplementary material and Supplemental (S.) Table 1 for details. All research was performed in accordance with relevant guidelines/regulations, and informed consent was obtained from all participants and/or their legal guardians.

### Genotyping

DNA was extracted from whole blood. We genotyped Cohort 1 on the Illumina Infinium Omni Express-24 v1.1 platform (Illumina Inc., San Diego, CA, USA) at deCODE Genetics (Reykjavik, Iceland) in concordance with the standard Illumina protocol. We genotyped Cohort 2 samples with the Illumina Infinium Omni Express Exome-8v1.3 chip. Cohort 3 samples were genotyped on Illumina’s HumanHAP300, HumanHAP300-Duo and HumanCNV370 bead arrays. We conducted assignment of genotypes according to the standard Illumina protocol in GenomeStudio software V2011.1 version 1.9.4. We tested for plate effects and other batch effects by a number of association tests described in detail under supplementary methods. Markers exhibiting high rates of genotyping missingness (above 5%), minor allele frequency (MAF) below 1% or showing departure from Hardy Weinberg equilibrium (p < 1 × 10^−4^ calculated for controls) were excluded from the analyses. Individuals showing high rates of genotyping missingness (above 5%), cryptic relatedness (pairwise Identity-By-Descent PI_HAT above 20%) or genome-wide heterozygosity (outside mean ± 5 SD of the sample) were removed from the analyses. Further, sex-check was performed based on the homozygosity estimate of X chromosome markers implemented in PLINK.

### Association analysis

We performed association analysis in two stages. Due to data regulations and ethical approvals regarding data sharing, we performed genome-wide association analyses on Cohorts 1 and 2 independently and combined the results through meta-analysis to obtain Stage 1 results. HRC imputation was not accessible for cohort 1 due to national regulations in Norway. To reduce possible genomic inflation or overcorrection, the results from the Stage 1 meta-analysis were corrected for genomic inflation before we performed meta-analysis with Cohort 3 to obtain Stage 2 results.

Genotypes from Cohort 1 samples were imputed onto the European reference haplotypes from the 1000 Genomes Project (GRCh37/hg19 assembly) Phase 3 using MACH (http://www.sph.umich.edu/csg/abecasis/MACH). We excluded variants with MAF lower than 0.01 or R-squared quality metric (INFO) > 0.5. We performed principal component analysis (PCA) on Cohort 1 pre-imputation data using PLINK 1.9 (https://www.cog-genomics.org/plink2) to account for population stratification. The association analysis by logistic regression on dosage data using PLINK 1.9 included gender, age and the two first principal components as covariates. Genomic inflation factors were calculated as the ratio of the median of the empirically observed distribution of the association chi-square statistic to the expected median^20^.

Genotypes from Cohort 2 samples were imputed onto the GRCh37/hg19 assembly with ShapeIT & Minimac3 using the haplotype reference consortium HRC version r1.1 reference data at the imputation server of the University of Michigan. PCA was done independently for Cohort 2 because there was no relatedness to samples from Cohort 1. Logistic regression was performed using PLINK 1.9 using gender, age and the top two genetic principal components as covariates. The genomic inflation factor was calculated as previously described.

To obtain Stage 1 results, variants from Cohort 1 and Cohort 2 were mapped to each other using GRCh37/hg19 assembly. All variants with allele discrepancies across cohorts were discarded. We performed meta-analysis of Cohort 1 and Cohort 2 using PLINK 1.9 with fixed effects inverse-variance weighted effect sizes. Biases from different cohorts due to genotype array and imputation procedures are mitigated through correction on the inflation factor. The results were verified using METAL meta-analysis tool (http://csg.sph.umich.edu/abecasis/Metal). To identify independently associated loci, we used FUMA’s SNP2GENE function to define lead SNPs and genomic risk loci^21^. Graphical representations including quantile-quantile plots and Manhattan plots were performed in R using the qqman package (http://cran.r-project.org/web/packages/qqman).

We selected variants with Stage 1 meta-analysis p-value < 1 × 10^−6^ for follow up in Cohort 3 (Iceland) and used the same approach described above to meta-analyse the results in Stage 2^22^.

### Functional mapping and annotation (FUMA) of GWAS

We utilized FUMA to functionally annotate our Stage 1 results^21^. FUMA incorporates 18 biological data repositories such as the Genotype-Tissue Expression (GTEx), the Encyclopedia of DNA Elements (ENCODE), the Roadmap Epigenomics Project and chromatin interaction information. FUMA requires GWAS summary statistics and its outputs include multiple tables and figures containing extensive information on, e.g., functionality of SNPs in genomic risk loci, including protein-altering consequences, gene-expression influences, open-chromatin states as well as three-dimensional (3D) chromatin interactions. Functionally annotated variants are subsequently mapped to prioritized genes based on (i) physical position mapping on the genome, (ii) expression quantitative trait loci (eQTL) mapping and (iii) 3D chromatin interactions (chromatin interaction mapping). Biological information for each prioritized gene is provided to gain insight into previously associated diseases. On top of the single gene level analyses, FUMA also provides information on association overrepresentation in sets of differentially expressed genes (DEG) to identify tissue specificity of prioritized genes. We refer to the details of methods and repositories of FUMA in^21^.

### Ethics committee approval

All cohorts and sites providing samples for this study have local ethics approval for DNA collection and data sharing, and the names of local ethics committees are provided in the in the supplemental materials. In Norway the joint study was approved by the Regional Committees for Medical and Health Research Ethics in Mid Norway.

## Results

**Figure 1:**
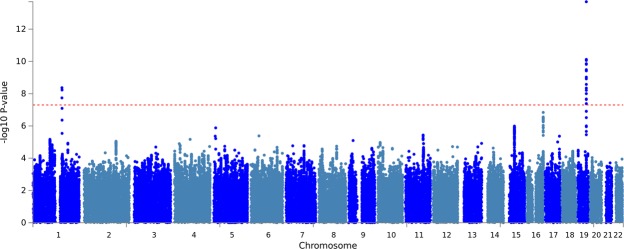
Manhattan plot of Stage 1 meta-analysis. Manhattan plot of meta-analysis of Cohorts 1 and 2 for genome-wide association with Dementia with Lewy Body (DLB). Genome-wide significant associations to DLB (threshold *P* < 5 × 10^−8^) are found in chromosomes 1 (*ASH1L/GBA*) and 19 (*APOE*), and a suggestive association to DLB at P < 1 × 10^−6^ is identified at chromosome 16 (*ZFPM1*). A comprehensive result of Stage 1 is presented in Supplementary Table 2.

From Cohort 1, we obtained genotypes for 719,755 SNPs and performed imputation to obtain 7,769,477 high-quality variants. We performed association using 478 DLB cases and 1322 controls, see S. Table 1. After controlling for population stratification using PCA (S. Fig. 1A), the genomic inflation factor Lambda was 1.005 (S. Fig. 2A). We found genome-wide significance on rs2230288 (closest gene *GBA*, p = 3.77 × 10^−8^) and rs429358 (closest gene *APOE*, p = 3.21 × 10^−9^). The regional association plots for this locus is visualized in S. Fig. 3.

To increase the power of our study, we included additional Norwegian population controls in the study, see S. Table 1. Using two principal components (S. Fig. 1B), the addition of population controls increased inflation to a Lambda of 1.244, possibly due to inflation from the additional controls. We verified the inflation using LD Score Regression and found the intercept at 1.2094, consistent with Lambda. Quantile-quantile plots for Cohort 1 before and after genomic correction are given in S. Fig. 2. After correction, the strongest associations in Cohort 1 remain with rs2230288 (p = 1.77 × 10^−10^) and rs429358 (p = 4.13 × 10^−9^).

We found 45 SNPs associated to DLB at p < 5 × 10^−6^ with strong associations in Chromosomes 1 and 19; a summary of our findings from Cohort 1 is given in S. Table 2A.

From Cohort 2, we analysed 7,570,659 successfully imputed variants. The genomic inflation factor Lambda was 1.031. Quantile-quantile plots for Cohort 2 are given in S. Fig. 2. The Cohort 2 study revealed 9 SNPs associated to DLB at p < 5 × 10^−6^; also with strong associations in Chromosome 19, see S. Table 2B. After individual analyses of discovery Cohorts 1 and 2, we performed a Stage 1 meta-analysis of 6,963,063 variants (898 were discarded due to allele mismatches). The meta-analysis genomic inflation factor Lambda was 0.865, possibly due to overcorrection for the genomic inflation in Cohort 1. We corrected the chi-square statistics of the meta-analysis at fixed ORs, see the quantile-quantile plots in S. Fig. 2. Genome-wide Stage 1 results are visualized as a Manhattan plot in Fig. 1.

After correction, Stage 1 analysis revealed 108 SNPs associated with DLB at p < 5 × 10^−6^ (S. Table 2C). The statistical power of our study is estimated to be 0.085 (MAF = 0.05) to 0.395 (MAF = 0.1) to 0.823 (MAF = 0.2) for SNPs with genomic risk ratio GRR = 1.5 and GRR = 1.2, shown in S. Fig. 4. GRR values were chosen based on ORs of discoveries of earlier DLB studies^13^. We followed up on 18 of these SNPs, which were successfully analysed in an independent sample from Iceland (Cohort 3) and performed a Stage 2 meta-analysis. Because stage 2 meta-analysis included only 18 selected SNPs instead of a genome-wide analysis, this result was not corrected for genomic inflation.

From the Stage 2 meta-analysis, we found two susceptibility regions associated with DLB surpassing genome-wide significance, p < 5 × 10^−8^. We found *APOE ε4* related SNPs at genome-wide significance, represented by rs429358 (OR = 2.28, p = 6.15 × 10^−17^, see Table 1 for details). A regional association plot of the *APOE* locus from Stage 1 meta-analysis is presented in S. Fig. 5A. From a recent large study, we found that this SNP is identical to the reported top hit (OR = 2.40, p = 1.05 × 10^−48^) in Guerreiro *et al*.^13^.

We also discovered a DLB-associated locus on Chromosome 1, represented by rs12734374 (closest gene: *ASH1L*, OR = 4.31, p = 1.33 × 10^−9^, see Table 1 and regional association plots in S. Fig. 5B). This SNP is located in the same genomic region of rs2230288, the strongest hit in Cohort 1 which was not successfully imputed in Cohort 2, but had implicated *GBA*. Furthermore, in another study, we found that rs12734374 is in high LD (R^2^ = 0.79) with a *GBA* hit, rs35749011 (OR = 2.27, p = 6.57 × 10^−10^ in)^13^. Both our *APOE* and *ASH1L*/*GBA* hits provide genome-wide significant confirmations of the findings from Guerreiro *et al*.^13^.

Furthermore, we investigated SNPs with a suggestive association to DLB. From the Stage 1 meta-analysis, we noted 9 SNPs at p < 1 × 10^−6^ in chromosome 16, represented by rs12926163 (closest gene *ZFPM1*, OR = 1.68, p = 1.45 × 10^−7^). These SNPs were not successfully analysed in the Icelandic cohort, and therefore we present only the Stage 1 result of this locus in Table 1 and S. Fig. 5C.

**Table 1:** Genetic loci with significant and suggestive associations with DLB at meta-analysis Stage 1 and Stage 2.

SNP	CHR:BP	Allele (min/maj)	MAF (1KG)	MAF Cohort 1	MAF Cohort 2	Gene	Meta Stage 1		Meta Stage 2	
							OR	P	OR	P
rs429358	19:45411941	C/T	0.155	0.143	0.153	*APOE e4*	2.79	2.00e-14	2.28	6.15e-17
rs12734374	1:155388851	T/A	0.023	0.022	0.012	*ASH1L*/*GBA*	4.29	4.29e-09	4.31	1.33e-09
rs12926163	16:88572056	C/T	0.311	0.252	0.323	*ZFPM1*	1.68	1.45e-07	—	—

CHR:BP: Chromosome and Base pair location based on Build 37, Assembly Hg19. Allele (min/maj): Minor and major alleles; MAF: Minor Allele Frequency (on European 1000 G), Cohort 1 and Cohort 2. Gene: Nearest gene within 500 kB; OR, P: case-control odds-ratio and association P-values from Stage 1 combining Cohorts 1 and 2, and Stage 2 Meta-analysis combining Cohorts 1, 2 and 3.

Next, we analysed specific gene signals reported previously in DLB for their significance under locus-wide Bonferroni correction for each gene. Due to the small SNP coverage in the Stage 2 analysis, we used results from Stage 1. We extracted variant information for *SNCA* (GRCh37hg19 chr4: 90,645,250-90,759,466), *SCARB2* (chr4:77,079,886-77,155,689), *MAPT* (chr17:43,971,748-44,105,700) and *CNTN1* (chr12:41,086,244-41,466,220) with upstream and downstream flanking of 200kB. Regional plots of these candidate genes are shown in S. Fig. 5D–G.

Among the 1509 successfully imputed SNPs in the *SNCA* locus, the strongest association was with rs2301135 (chr4:90,758,389, p = 5.68 × 10^−5^, OR = 1.40, minor allele C) and remained nearly significant after correction (threshold p < 3.3 × 10^−5^) using conservative multiple test assumptions of independent SNPs. In the *SCARB2* locus, the strongest association among the 1600 SNPs was with rs34216031 (chr4:76,971,832, OR = 1.63, p = 1.37 × 10^−2^), but its significance did not survive correction (threshold p < 3.1 × 10^−5^). In the *MAPT* locus, 694 SNPs passed quality checks. Among these, the strongest association was with rs11652003 (chr17:44,132,659, OR = 0.75, p = 1.89 × 10^−2^) but did not withstand correction (threshold p < 7.2 × 10^−5^). Of note, coverage of *MAPT* is relatively poor in our genotyping and imputation procedure, see S. Fig. 5F. In the *CNTN1* locus, the strongest association was with rs56260639 (chr12:41122583, OR = 0.50, p = 1.17 × 10^−2^). Despite the strong OR, this association did not remain significant after correction (threshold p < 2.1 × 10^−5^).

Finally, we investigated the potential biological roles of the resulting list of genes in brain disorders. For this, we performed functional analysis with FUMA GWAS^21^. We summarized the independent genomic risk loci from Stage 1 with suggestive association p < 1 × 10^−6^ in S. Fig. 6. The strongest associations were close to *APOE* in Chromosome 19 and distributed in a relatively small region spanning only 41kB and 4 genes. The significant associations in Chromosome 1, within the large haploblock containing *GBA*, spanned 1.2MB and up to 64 genes (see S. Fig. 6). We computed gene-based P-value test for protein-coding genes by mapping SNPs to genes if SNPs were located within the genes. The chromosome 1 genes (*GBA*) did not surpass the significance threshold (S. Fig. 7), but the chromosome 19 genes (*APOE*, *APOC1*, *TOMM40*) remained significant. We also found significant results applying the gene-based test to the gene on chromosome 16 (*ZPFM1*), which was suggestive at the single variant level. Using MAGMA tissue expression analysis, we found the strongest expression in whole blood, substantia nigra and spinal cord cervical level c-1. (S. Fig. 8).

FUMA prioritized 65 genes (S. Table 3, S. Fig. 9) for further functional analyses; see Methods on how genes are prioritized. Of note, in Chromosome 1, *GBA* was prioritized based on eQTL analysis (S. Fig. 9), further strengthening the case that our top hit implicated not only *ASH1L* but also *GBA*. From the set of 65 genes, we looked up tissue specific expression patterns based on GTEx v6 RNA-seq data. These are visualized as a heatmap in S. Fig. 10 ^21^. Relative to other genes, we found *APOE* highly expressed in all tissues (S. Fig. 10A). *ASH1L* and *GBA* are moderately expressed and *ZFPM1* has a lower gene expression relative to other genes in all tissues. Next, we looked at the tissue specificity for each gene. We found *APOE* with moderately higher expression in brain tissue (S. Fig. 10B), while *ASH1L*, *GBA* and *ZFPM1* are not specific to brain tissues. Notably, we found higher expression in brain tissues for *PAQR6*, *CHRNB2*, *SYT11* and *APOC1* and conversely we found lower expression for *PVRL2*, *LMNA* and *SHC1* (S. Fig. 10B).

Besides the single gene level analyses, we also identified tissue specificity of prioritized genes by looking at overrepresentation in sets of differentially expressed genes (DEG), see S. Fig. 11. DEG for each tissue was calculated in FUMA. We found the spinal cord cervical level c-1 and amygdala being two of the top five tissues with the most DEG, however, none passed Bonferroni corrected significance. The finding of the spinal cord cervical level c-1 is consistent with the MAGMA analysis.

## Discussion

We performed a genome-wide association study based on 828 clinically diagnosed DLB cases and a large sample of 82035 controls. We confirmed the *APOE ε4* allele and a locus close to *ASH1L* and *GBA* (Chr1q22) as significantly associated with DLB. Furthermore, we nominate a novel genetic locus near *ZFPM1* as suggestively associated with DLB. Taken together with recent findings from another DLB GWAS^13^, the current results firmly establish *APOE e4*, *SNCA* and *GBA* as robust risk loci for DLB, which implicate novel disease mechanisms to be followed up in experimental studies.

The top-hit SNP at the 1q22 locus is located within the large haploblock containing the *GBA* (glucocerebrosidase gene), also recently identified by Guerreiro *et al*.^13^. We note that the strongest association was in SNPs with relatively high LD (D′ 0.66) with rs2230288, referred to in the literature as the *GBA* E326K or 365 K polymorphism. E326K is a low frequency coding variant, which unlike the “neuropathic” *GBA* mutations does not cause Gaucher’s disease in the homozygous state. We recently demonstrated that this variant accounts for the *GBA* top-hit from a PD meta-GWAS^23^. E326K has also been associated with worse cognitive outcomes in PD^15,16^. We inspected the association results further and found that E326K showed the strongest of all associations at this locus in Cohort 1, yet was not successfully imputed in Cohort 2. We thus consider it likely that E326K is the functional variant underlying this signal. With an allele frequency of >2% in the population and a strong effect on susceptibility to both PD and DLB, this variant emerges as a major risk factor for the Lewy body disorders combined. From a functional perspective, the *GBA* association highlights the importance of lysosomal pathways in DLB pathogenesis. *GBA* was recently confirmed in the largest GWAS in DLB to date (1743 DLB patients included) as the third most strongly associated risk gene^13^. *SCARB2 (scavenger receptor class B member 2)*, encoding another lysosomal enzyme, has previously been associated with DLB^12^. While *GBA* is probably the most plausible causative gene in the 1q22 locus, we cannot rule out other candidate gene such as *ASH1L* (Absent, Small or Homeotic discs 1-Like). The gene encodes a histone-lysine N-methyltransferase, a member of the trithorax transcriptional regulators which are essential for development, organ function and fertility.

We and others have previously reported *APOE (Apolipoprotein E) ε4* (Chr19q13.32) as an important genetic risk factor for DLB. Guerreiro *et al*. found the locus highly significant^13^, and we reported an OR for carriers of one copy of the *APOE ε4* allele to be 2.9 for developing DLB and 4.2 for developing AD. For carriers of two copies of the *APOE ε4* alleles the OR for developing DLB increased to 5.9 while the OR for developing AD was as high as 15.2^14^. Bras and colleagues have reported *APOE* as the strongest associated risk gene in both clinically and neuropathologically diagnosed DLB cases^12^, and this was confirmed in an expanded cohort from the same group recently^13^. Guerreiro *et al*. estimated the DLB SNP-heritability based on the Illumina Neuro-X content to be 31%, with *APOE* accounting for about 9%^11^. *APOE ε4* has also been found to increase the risk of dementia in pure alpha-synucleinopathies in a study where its frequency was 38% in pathologically diagnosed pure AD, 40.6% in the mixed AD and DLB group, 31.9% in pure DLB, 19.1% in Parkinson’s Disease Dementia (PDD) and 7.2% among healthy controls^24^. In another AD GWAS, Lewy body pathology in the brain was associated with *APOE* variants^25^. Most cases with clinically diagnosed DLB will contain both Lewy bodies and AD pathology in the brain^7^.

*SNCA (synuclein alpha)* is the strongest associated risk gene in PD and encodes α-synuclein, which is a major constituent of Lewy bodies, pathological hallmark for both DLB and PD/PDD. Accumulation of α-synuclein aggregates have been found to create synaptic dysfunction in DLB^26^. The top associated variant in our data (rs2301135) is in LD with the *SNCA* signal reported as significant in the previous study by Bras *et al*. (r-squared 0.98 and D′ 1.0 with rs894280 in 1000 genomes European population)^12^ and the secondary signal from a large meta-analysis of PD GWAS (r-squared 0.98 and D′ 1.0 with rs7681154)^27^. Both p-value and effect size of the *SNCA* association observed here are equivalent to those found in the similarly sized DLB study by Bras *et al*.^12^, and despite falling short of genome wide significance, we interpret this result as supportive for an *SNCA* association in DLB. Deviations from other studies with respect to the strongest SNP at the locus could well arise if key SNPs are not well imputed across all cohorts.

Together, the identified genetic loci could be involved in a common neurobiological disease pathway in DLB. The normal degradation of α-synuclein is highly dependent on lysosomal function and glucocerebrosidase is an important enzyme in this degradation. Impaired function of glucocerebrosidase due to coding variants like E326K will slow down the degradation of α-synuclein thus increasing the concentration of toxic oligomers and thereby driving the pathological process in DLB. Inhibition of lysosomal enzymes also results in Aβ accumulation and aggregation. The apolipoproteins accelerate neuronal Aβ uptake, lysosomal trafficking and degradation in an isoform-dependent manner with apolipoprotein E3 more efficiently facilitating Aβ trafficking and degradation than apolipoprotein E4, a risk factor for AD and DLB^28^, thus linking both *GBA*, *APOE* and *SNCA*.

The present findings of genetic loci suggestively associated with DLB indicate interesting pathological mechanisms. The chromosome 16 locus associated with DLB at p = 1.45 × 10^−7^ implicates *ZFPM1* (Zinc finger protein, FOG family member 1), which is expressed in human hematopoietic tissues and in the cerebellum and is involved in erythroid differentiation. In one study of AD and psychosis, duplications in this gene were associated with psychosis in AD, a symptom highly relevant in DLB, were visual hallucinations and related delusions are core symptoms of the disease^29^. Our findings suggest other putative molecular mechanisms in DLB.

*CNTN1* (contactin 1) is a glycosylphosphatidylinositol anchored neuronal membrane protein that functions as a cell-adhesion molecule with important roles in axonal function. It is located near the *LRKK2* locus and was associated to PD and reported as a suggestive hit in the largest GWAS of DLB to date^13^. We found no significant hits under correction in our study. Further, we found no genome-wide significant associations with *MAPT (microtubule associated protein tau)*, the gene encoding tau, in our study. However, this gene was poorly covered in our genotyping and imputation procedure. *MAPT* is the second strongest associated risk gene in PD and is associated also with AD^30^. It exists on two different haplotypes, H1 and H2. H1P has been associated with Parkinson’s disease with dementia, whereas H1C has been associated with Alzheimer’s disease. Thus, *MAPT* would be a plausible candidate gene also for DLB due to clinical and likely genetic overlap with AD and PD beyond *APOE*^31,32^. The negative finding suggests that the role of *MAPT* variability might represent a genetic difference between DLB and PD, but this hypothesis needs to be further tested in larger cohorts, preferentially including both DLB and PD samples.

All cases included in our study were clinically diagnosed with common sporadic and late onset type of DLB. Cases solely diagnosed based on pathology might not always fulfil clinical diagnostic criteria during life, and therefore might include cases of PD and even early onset PD developing dementia in late stages. The clinically diagnosed DLB cases included in our study might therefore have a purer, less PD-like genetic profile than studies based on brain bank cases, a possible explanation for why we do not find the previously PD-associated risk loci like *MAPT* in our analysis. Diagnosing DLB clinically is challenging both because of the clinical heterogeneity and the overlapping AD pathology masking typical DLB core symptoms in many cases. Although diagnostic procedures differed among centres, nearly all centres are academic dementia research centres with high-level clinical expertise on DLB and used standardized and established procedures, including, in a subset of patients, biomarkers. Thus, we believe diagnoses were as accurate as can be achieved in a clinical setting, although pathological confirmation was available only in a subset. The clinical diagnostic criteria for probable DLB have been found to have high specificity and this was confirmed in a pathological study in one of the samples included in this study^7^. We therefore argue that only few cases with other diagnoses like AD can have been erroneously included in this sample. Adding biomarkers like (123)-FP-CIT-SPECT to the diagnostic procedure could increase diagnostic precision in DLB. AD-pathology could be detected by PET or CSF-biomarkers of amyloid and tau deposition^33^. The recently published revised diagnostic criteria for DLB are slightly different from the previous^1^. Whether this will impact on the genetic architecture of DLB cohorts is not known, however.

There are few other large cohorts diagnosed with DLB with well characterized patients, and although this is one of the largest studies in DLB to date – sample size is still small for a hypothesis-free GWAS approach. Consequently, we only had statistical power to detect signals with large effect sizes, such as *APOE* and *GBA*. We anticipate that GWAS with larger samples will detect more common genetic risk loci associated with DLB with effect sizes comparable to the vast majority of AD and PD GWAS loci. Current evidence further indicates that rare variants contribute significantly to the disorder, suggesting next generation sequencing approaches will also be important to further characterize the genetic architecture of DLB.

DLB is increasingly recognized as a specific clinical diagnosis distinct from AD and PD both clinically and genetically, and has a poor prognosis with no approved treatment. To detect more of the genetic risk loci contributing to DLB pathogenesis new methods like Bayesian statistics may prove useful. This notwithstanding, larger samples obtainable through international collaboration are needed in a future GWAS of DLB. We therefore plan to collaborate to increase sample size in a next step to increase the power to detect more common genetic variants with small effects associated with the risk of development of DLB.

## Supplementary information

Supplemental material

## Data Availability

Genotype datasets from the Norwegian DemGene network generated and analysed during the current study are not publicly available due to compliance to privacy. Summary statistics are available from the corresponding author on reasonable request.
