# Author Correction: *GBA* and *APOE ε4* associate with sporadic dementia with Lewy bodies in European genome wide association study

**DOI:** 10.1038/s41598-019-51827-0

**Published:** 2019-10-17

**Authors:** Arvid Rongve, Aree Witoelar, Agustín Ruiz, Lavinia Athanasiu, Carla Abdelnour, Jordi Clarimon, Stefanie Heilmann-Heimbach, Isabel Hernández, Sonia Moreno-Grau, Itziar de Rojas, Estrella Morenas-Rodríguez, Tormod Fladby, Sigrid B. Sando, Geir Bråthen, Frédéric Blanc, Olivier Bousiges, Afina W. Lemstra, Inger van Steenoven, Elisabet Londos, Ina S. Almdahl, Lene Pålhaugen, Jon A. Eriksen, Srdjan Djurovic, Eystein Stordal, Ingvild Saltvedt, Ingun D. Ulstein, Francesco Bettella, Rahul S. Desikan, Ane-Victoria Idland, Mathias Toft, Lasse Pihlstrøm, Jon Snaedal, Lluís Tárraga, Mercè Boada, Alberto Lleó, Hreinn Stefánsson, Kári Stefánsson, Alfredo Ramírez, Dag Aarsland, Ole A. Andreassen

**Affiliations:** 1 Haugesund Hospital, Helse Fonna, Department of Research and Innovation, Haugesund, Norway; 2 The University of Bergen, Department of Clinical Medicine (K1), Bergen, Norway; 3 NORMENT, KG Jebsen Centre for Psychosis Research, Division of Mental Health and Addiction, Oslo University Hospital, Oslo, Norway; 4 Institute of Clinical Medicine, University of Oslo, Oslo, Norway; 5 Memory Clinic and Research Center of Fundació ACE, Institut Català de Neurociències Aplicades, Universitat Internacional de Catalunya (UIC), Barcelona, Spain; 6 Department of Neurology, II B Sant Pau, Hospital de la Santa Creu i Sant Pau, Universitat Autònoma de Barcelona, Barcelona, Spain; 7 Center for Networker Biomedical Research in Neurodegenerative Diseases (CIBERNED), Madrid and Barcelona, Spain; 8 Institute of Human Genetics, University of Bonn, Bonn, Germany; 9 Department of Genomics, Life & Brain Center, University of Bonn, Bonn, Germany; 10 Department of Neurology, Akershus University Hospital, Lørenskog, Norway; 11 University of Oslo, AHUS Campus, Oslo, Norway; 12 Department of Neuromedicine and Movement Science, Norwegian University of Science and Technology, Trondheim, Norway; 13 Department of Neurology, St Olav’s Hospital, Trondheim, Norway; 14 University Hospital of Strasbourg, CMRR (Memory Resources and Research Centre), Geriatrics Department, Strasbourg, France; 15 University of Strasbourg and CN RS, ICube laboratory and FMTS, team IMIS/Neurocrypto, Strasbourg, France; 16 University Hospital of Strasbourg, CMRR (Memory Resources and Research Centre), Laboratory of Biochemistry and Molecular Biology, Strasbourg, France; 17 University of Strasbourg and CNRS, Laboratoire de Neurosciences Cognitives et Adaptatives (LNCA), UMR7364, 67000 Strasbourg, France; 18 Alzheimercenter & Department of Neurology VU University Medical Center, Amsterdam, The Netherlands; 19 Lund University, Skane University Hospital, Institute of Clinical Sciences, Malmö, Sweden; 20 Department of Medical Genetics, Oslo University Hospital, Oslo, Norway; 21 NORMENT, KG Jebsen Centre for Psychosis Research, Department of Clinical Science, University of Bergen, Bergen, Norway; 22 Department of Psychiatry, Namsos Hospital, Namsos, Norway; 23 Department of Mental Health, Norwegian University of Science and Technology, Trondheim, Norway; 24 Department of Geriatrics, St. Olav’s Hospital, Trondheim, Norway; 25 Department of Geriatric Psychiatry, Oslo University Hospital, Oslo, Norway; 26 Departments of Radiology and Biomedical Imaging, Neurology and Pediatrics, UCSF, San Francisco, USA; 27 Oslo Delirium Research Group, Department of Geriatric Medicine, Institute of Clinical Medicine, University of Oslo, Oslo, Norway; 28 Research Group for Lifespan Changes in Brain and Cognition, Department of Psychology, University of Oslo, Oslo, Norway; 29 Institute of Basic Medical Sciences, University of Oslo, Oslo, Norway; 30 Department of Neurology, Oslo University Hospital, Oslo, Norway; 31 Landspitali University Hospital, Reykjavik, Iceland; 32 DeCODE genetics, Reykjavik, Iceland; 33 Division for Neurogenetics and Molecular Psychiatry, Department of Psychiatry and Psychotherapy, Medical Faculty, University of Cologne, 50924 Cologne, Germany; 34 Department for Neurodegenerative Diseases and Geriatric Psychiatry, University of Bonn, 53127 Bonn, Germany; 35 Institute of Psychiatry, Psychology and Neuroscience, King’s College London, London, UK; 36 Center for Age-Related Diseases, Stavanger University Hospital, Stavanger, Norway

Correction to: *Scientific Reports* 10.1038/s41598-019-43458-2, published online 07 May 2019

The Acknowledgements section in this Article is incomplete.

“The authors would like to thank the Norwegian Dementia Genetics Network (DemGene), the European DLB Consortium (E-DLB), Dementia Genetics Spanish Consortium (DEGESCO) and Genomic Research at Fundació ACE (GR@ACE) Consortium. Fundació ACE would like to thank patients and controls who participated in this project. We are indebted to private donors for their support of Fundació ACE research programs. Fundació ACE collaborates with the Centro de Investigación Biomédica en Red sobre Enfermedades Neurodegenerativas (CIBERNED, Spain) and is one of the participating centers of the Dementia Genetics Spanish Consortium (DEGESCO). The study was founded by the Research Council of Norway, Norwegian Regional Health Authorities and Norwegian Health Association and 237250/EU/JPND (APGeM). The Cohort 2 results were generated with the assistance of financial support of grants PI13/02434 and PI16/01861. Acción Estratégica en Salud, integrated in the Spanish National R + D + I Plan and financed by ISCIII (Instituto de Salud Carlos III)-Subdirección General de Evaluación and the Fondo Europeo de Desarrollo Regional (FEDER- “Una manera de Hacer Europa”). GR@ACE project has been funded by Fundación bancaria “La Caixa”, Grifols SA, Fundació ACE. The funding sources did not in any way affect the results or presentation of the results.”

should read:

“The authors would like to thank the Norwegian Dementia Genetics Network (DemGene), the European DLB Consortium (E-DLB), Dementia Genetics Spanish Consortium (DEGESCO) and Genomic Research at Fundació ACE (GR@ACE) Consortium. Fundació ACE would like to thank patients and controls who participated in this project. We are indebted to private donors for their support of Fundació ACE research programs. Fundació ACE collaborates with the Centro de Investigación Biomédica en Red sobre Enfermedades Neurodegenerativas (CIBERNED, Spain) and is one of the participating centers of the Dementia Genetics Spanish Consortium (DEGESCO). The study was founded by the Research Council of Norway, Norwegian Regional Health Authorities and Norwegian Health Association and 237250/EU/JPND (APGeM). The Cohort 2 results were generated with the assistance of financial support of grants PI13/02434 and PI16/01861. Acción Estratégica en Salud, integrated in the Spanish National R + D + I Plan and financed by ISCIII (Instituto de Salud Carlos III)-Subdirección General de Evaluación and the Fondo Europeo de Desarrollo Regional (FEDER- “Una manera de Hacer Europa”). GR@ACE project has been funded by Fundación bancaria “La Caixa”, Grifols SA, Fundació ACE. The funding sources did not in any way affect the results or presentation of the results. This paper represents independent research partly funded by the National Institute for Health Research (NIHR) Biomedical Research Centre at South London and Maudsley NHS Foundation Trust and King’s College London. The views expressed are those of the author and not necessarily those of the NIHR or the Department of Health and Social Care.”

