# Supplementary material for: GBA and APOE ε4 associate with sporadic dementia with Lewy bodies in European genome wide association study
Source: Sci Rep. 2019 May 7;9:7013. doi: 10.1038/s41598-019-43458-2 (PMC6504850; doi:10.1038/s41598-019-43458-2)
Supplement: Supplementary file 1 — Supplemental material [file 41598_2019_43458_MOESM1_ESM.docx]

**GBA and APOE ε4 associate with sporadic dementia with Lewy bodies in European genome wide association study**

Authors: Arvid Rongve*, Aree Witoelar*, Agustín Ruiz, Lavinia Athanasiu , Carla Abdelnour, Jordi Clarimon, Stefanie Heilmann-Heimbach, Isabel Hernández, Sonia Moreno-Grau, Itziar de Rojas, Estrella Morenas-Rodríguez, Tormod Fladby, Sigrid B. Sando, Geir Bråthen, Frédéric Blanc, Olivier Bousiges, Afina W. Lemstra, Inger van Steenoven, Elisabet Londos, Ina S. Almdahl, Lene Pålhaugen, Jon A. Eriksen, Srdjan Djurovic, Eystein Stordal, Ingvild Saltvedt, Ingun D. Ulstein, Francesco Bettella, Rahul S Desikan, Ane-Victoria Idland, Mathias Toft, Lasse Pihlstrøm, Jon Snaedal, Lluís Tárraga, Mercè Boada, Alberto Lleó, Hreinn Stefánsson, Kári Stefánsson, Alfredo Ramírez, Dag Aarsland and Ole A. Andreassen.

*Contributed equally

**SUPPLEMENTARY MATERIALS**

[Supplemental Text 1. Methods 2](#_Toc532902150)

[Supplemental Table 1: DLB and control cohorts 8](#_Toc532902151)

[Supplemental Table 2: Top SNP associations with DLB 9](#_Toc532902152)

[Supplemental Table 3: FUMA prioritized genes from Stage 1 meta-analysis 13](#_Toc532902153)

[Supplemental Figure 1. Cohort 1 Principal Component Analysis (PCA) plots 15](#_Toc532902154)

[Supplemental Figures 2: Stage 1 Quantile-quantile (Q-Q) plots 16](#_Toc532902155)

[Supplemental Figures 3: Regional locus association plot of Cohort 1 without population controls 21](#_Toc532902156)

[Supplemental Figure 4. Statistical Power of DLB GWAS 23](#_Toc532902157)

[Supplemental Figures 5: Regional locus association plots of Meta-Analysis Stage 1 25](#_Toc532902158)

[Supplemental Figure 6: FUMA GWAS Summary 32](#_Toc532902159)

[Supplemental Figure 7: Gene-based Manhattan plots 33](#_Toc532902160)

[Supplemental Figure 8: MAGMA Tissue Expression Analysis 34](#_Toc532902161)

[Supplemental Figure 9: Circos plots of chromatin interactions and eQTLs 35](#_Toc532902162)

[Supplemental Figure 10: Gene expression heatmap 38](#_Toc532902163)

[Supplemental Figure 11: Differentially expressed genes across 53 specific tissue types 40](#_Toc532902164)

# Supplemental Text 1. Methods

**Cohorts**

*Cohorts 1&2.* We collected genotype data for Cohort 1 from the Norwegian Dementia Genetics Network (DemGene) ^1^ and from the European DLB consortium (E-DLB) ^2^. Due to privacy restrictions, we assign our discovery data from DemGene and three European centres (Strasbourg, Amsterdam and Lund) as Cohort 1 and two European centres (Barcelona) as Cohort 2.

In Cohort 1, the Norwegian DLB samples (n=259) came from 6 study sites: the AHUS study (*n* = 16) ^3^, the Dementia Study of Western Norway (DemVest)(*n* = 55) ^4^, the Progression of Alzheimer’s Disease and Resource Use study (PADR) (*n* = 5), the Norwegian Register of Cognitive Symptoms (NorCog) (*n* = 43) ^5^, HUNT Health and Memory Study (n=36) ^6^ and the TrønderBrain study (*n* = 104) ^7^. The European samples (n=223) were collected as part of the E-DLB consortium consisting of European centres with a special expertise in DLB ^8^ and came from The AlphaLewyMA study from Strasbourg, France (n=60) ^9^, the Amsterdam Dementia Cohort, The Netherlands (n=125) ^10^ and Lund, Sweden (n=38). A total of 1760 control subjects without dementia from Norway were included in Cohort 1: the AHUS study (*n* = 82) ^3^, DemVest (n=2), the PADR study (*n* = 1), the Dementia Study in Rural Northern Norway (*n* =196) ^11^, the TrønderBrain study (*n* = 725) ^7^, the HUNT study (*n =*146) ^6^, The COGNORM study (n = 100) ^12^, The Oslo Parkinson’s disease Cohort Study (n = 483) ^13^ and DDI (n=25) ^14^. To increase the statistical power of our association analysis, the controls were combined with additional 4992 population controls from Norwegian blood donor samples from Oslo University Hospital, Ullevål Hospital, Norway. Detailed descriptions on all cohorts are listed on the supplemental material.

In Cohort 2, we performed GWAS on 242 DLB cases and 297 controls from Sant Pau Hospital ^15^ and the Barcelona Alzheimer Treatment & Research Center (ACE) ^16^.

*Cohort 3.* The samples came from the Memory Clinic, Landspitali University Hospital, Iceland with 108 DLB cases ^17^ and 75545 controls ^18^.

**Diagnoses**

We diagnosed dementia according to the ICD-10 research criteria ^19^ or the DSM-IV criteria ^20^. Diagnostic procedures varied somewhat across centers, regarding the use of diagnostic and clinical rating scales. At all centers, however, a diagnosis of DLB was made according to the consensus criteria (McKeith 1996 or 2005 criteria) ^21,22^ after a thorough medical examination by a trained specialist in psychiatry, neurology or geriatric medicine. Standardized diagnostic scales like the MAYO sleep questionnaire and the MAYO fluctuation scale and the UPDRS part 3 motor symptoms were used in some of the cohorts ^4,5^. In addition, routine blood tests and structural brain imaging were used to exclude alternative diagnoses. In some patients, dopamine transporter (CIT-)SPECT and CSF analyses were performed to confirm clinical diagnosis ^23^. In a small subset of 20 participants, we confirmed the diagnosis postmortem by a standardized neuropathological diagnostic process by a trained and experienced neuropathologist ^24^. Additional details regarding the recruitment and diagnostic procedures in the different cohorts can be found below.

**Genotyping**

We evaluated plate effects by coding case/control status by plate followed by running GWAS testing on each plate against all other plates. Any single plate with mean P value deviating from 0.5 was excluded as well as plates with many more or fewer significant results. We performed a standard pre-imputation quality control on each batch separately followed by the same quality control protocol on the combined dataset with the addition of a control for batch effects across batches. The latter consists in a set up similar to that of the control for plate effects but removes markers showing association below FDR=0.9

**Detailed description of cohorts**

**Norwegian Study Groups**

The Norwegian Register of Cognitive Symptoms (NorCog) includes patients diagnosed with mild dementia or mild cognitive impairment referred to Memory Clinics. Recruitment began in the South-Eastern part of Norway and is now including most Memory Clinics in Norway. In the current study patients from the Memory Clinics at Oslo University Hospital, Haugesund Hospital, St. Olav’s Hospital and Innlandet Hospital are included. Dementia and cognitive impairment are diagnosed according to the ICD-10 research criteria and DLB according to the revised DLB criteria from 2005. Patients are home-dwelling outpatients referred to memory clinics, and had standardized comprehensive assessment, including a medical history from the patient as well as a close family member, comprehensive neuropsychological testing, a physical and psychiatric examination with the use of standardized assessment scales, blood sample analyses, CSF when appropriate and structural brain imaging with MRI in most cases and additionally amyloid-PET in selected cases. Ethical approvals were given from the Regional Committees for Medical and Health Research Ethics in Southeast and Western Norway.

The Progression of Alzheimer’s Disease and Resource study (PADR) recruited 222 patients at the outpatient clinic at the Department of Geriatric Medicine, St. Olav’s Hospital. These were home-dwelling patients assessed for cognitive impairment according to the same protocol used in the NorCog study. Mainly patients diagnosed with Alzheimer’s disease (both mild cognitive impairment and dementia) were recruited. Ethical approval was given from the Regional Committees for Medical and Health Research Ethic in Mid Norway.

The HUNT Health and Memory Study consists of two populations, a hospital population and a nursing home population. The hospital population recruited cases from the Nord-Trøndelag county hospital dementia register by a two-step procedure. First, a computerized search of the administrative patient database of the two hospitals in Nord-Trøndelag (Namsos Hospital and Levanger Hospital) identified patients who were referred for assessment of cognition at the outpatients’ clinics in the period between 1995 and 2010. Next, the hospital records of all such patients were reviewed by a diagnostic team of researches and clinical experts in dementia, consisting of a geriatrician and two geriatric psychiatrists. All included cases were diagnosed in two steps, first applying the clinical ICD-10 criteria for dementia; secondly, DLB was diagnosed in cases with two of the following three symptoms: fluctuating cognition, visual hallucinations and motor features of Parkinson’s disease.

The nursing home population recruited cases form a study of all patients in all nursing homes in Nord-Trøndelag County between June 2010 and March 2011. Dementia was assessed by interview with a professional caregiver using a questionnaire for identifying dementia symptoms developed for this study, as well as the Clinical Dementia Rating Scale (CDR). Cognitive function was assessed by two tests: the Mini-Mental-State Examination and the Severe Impairment Battery-8, and by interview with the caregiver using the Informant Questionnaire on Cognitive Decline in the Elderly (IQ-CODE). In addition, two physicians with clinical and research experience independently diagnosed mild cognitive impairment, the dementia syndrome and dementia subtypes, using all available information. More information about the cohort and the method used are described in details in. Ethical approval was given from the Regional Committees for Medical and Health Research Ethic in Mid Norway.

In the Dementia Study of Western Norway (DemVest study) all referrals to memory clinics in Hordaland and Rogaland counties were screened for a first time diagnosis of mild dementia. Patients diagnosed for the first time with a dementia diagnosis were offered inclusion. To select patients with mild dementia only, all subjects were screened with the Mini-Mental State Examination and a minimal score of 20 and CDR = 1 was required for inclusion. After the initial inclusion period (2005-2007), only DLB or PDD patients were included. Patients without dementia or with acute delirium or confusion, terminal illness, recently diagnosed with a major somatic illness, previous bipolar disorder or psychotic disorder were excluded from the study. A research clinician performed a structured clinical interview of demographics, previous diseases, and drug history of patients and caregivers. The assessment procedure included a detailed history using a semi structured interview, clinical examination including physical, neurological, psychiatric, and a detailed neuropsychological test battery, routine blood tests, spinal fluid from a subgroup and MRI scans. Dopamine transporter SPECT was available for most patients with suspected DLB. Patients were followed annually, and diagnoses were re-evaluated with regular intervals by a consensus panel based on all available data. DLB was diagnosed pathologically for 18 cases. Sensitivity, specificity, positive predictive value and negative predictive values were 83%, 93%, 88% and 90% for Lewy body dementias, and for probable DLB alone they were 77%, 94%, 83% and 91%. Ethical approval was given from the Regional Committees for Medical and Health Research Ethic in Western Norway.

In the AHUS study, case samples were taken from the biobank at Akershus University Hospital in July 2013 and included all samples of EDTA blood stored in the biobank from patients with cognitive complaints referred to lumbar puncture as part of their clinical work-up at the memory clinic. In addition, subjects participating in research projects on mild cognitive impairment and on Parkinson’s disease were included. Control subjects without cognitive complaints or known cerebral pathology were included, comprising samples from orthopedic patients and spouses of patients with cognitive impairment. The medical records of all participating patients were examined retrospectively and the final diagnosis was determined based on all available information. Patients were diagnosed according to established diagnostic criteria. Ethical approval was given from the Regional Committees for Medical and Health Research Ethic in Southeast Norway.

The Dementia Study in Rural Northern Norway recruited patients with recently diagnosed AD in primary health care in nine rural municipalities in Northern Norway from January 2006 to December 2007. AD patients were recruited from general practice and through population based screening. Both groups underwent similar cognitive, physical and laboratory examinations. Inclusion criteria were individuals aged ≥ 65 years with a MMSE sum score ≥ 10 points. Exclusion criteria were delirium and behavioral disturbances interfering with cognitive and clinical testing, reluctance to participate, and inability to understand the purpose of the study, or relatives/caregivers disapproving participation. Participating clinicians were trained to identify and diagnose AD based on the Norwegian guidelines. In addition to a semi-structured interview of the participants focusing on impairment of cognition and activities of daily living, the Informant Questionnaire on Cognitive Decline in the Elderly (IQCODE) was completed, and cognition was tested using the Mini Mental Status Examination (MMSE) and Clock Drawing Test. Depression was examined with Montgomery and Aasberg Depression Rating Scale. Neurological examination, blood tests and structural brain imaging (CT) were performed. The diagnosis of dementia was set by the GP and discussed with at least one specialist in geriatric medicine according to the ICD-10 criteria as previously described. Disagreement or uncertainty about the diagnostic subtypes regarding 12 patients was solved by consulting a third specialist in geriatric medicine. The controls were healthy age-matched individuals with normal cognitive function. Ethical approval was given from the Regional Committees for Medical and Health Research Ethic in Northern Norway.

In the TrønderBrain-study patients were recruited through the University Hospital in Trondheim, the district hospital in Namsos, nursing homes and local care authorities in central Norway. Patients or suitable proxies were asked about case history, including a family history of dementia. Neurological examination was completed by one single neurologist and included the Mini Mental State Examination and the Clock Drawing Test. Blood screening was performed and secondary causes of dementia were excluded. Additional tests including EEG, MRI and lumbar puncture were performed in subgroups. Control subjects were caregivers not genetically related to the patients and other elderly volunteers recruited from societies for retired people in central Norway. All controls were without first-degree relatives with dementia, were healthy for their age and displayed no signs of a neurological disorder. They were age and sex-matched to the patient groups as closely as possible. For both patients and controls only ethnic Norwegians were included. Ethical approval was given from the Regional Committees for Medical and Health Research Ethic in Mid Norway.

The COGNORM study consists of patients scheduled for elective gynecological (genital prolapse), urological (benign prostate hyperplasia, prostate cancer or bladder tumor/cancer) or orthopedic (knee or hip replacement) surgery in spinal anesthesia turning 65 years or older the year of inclusion. They were recruited from Oslo University Hospital and Diakonhjemmet Hospital in Oslo from February 2012 to June 2013. 172 subjects underwent a multi-domain cognitive assessment before the surgery, comprising the Mini Mental Status Examination (MMSE), Clock Drawing Test, Word List Memory Task, Trail Making Test A and B, Kendrick Object Learning Test, and verbal fluency (FAS test and Animal Naming). Informant Questionnaire on Cognitive Decline in the Elderly was filled out by a relative or friend. Blood and CSF were collected in conjunction with anesthesia, and brain MRI was taken during the months after surgery. Blood samples, CSF samples and brain MRIs were obtained from 163, 155 and 128 subjects, respectively. The subjects were followed with annual cognitive testing since baseline, and at two-year follow up a new brain MRI was obtained from 111 subjects. At the ongoing four-year follow-up a third MRI, an fMRI, and a second lumbar punction for CSF sampling is performed. Annual follow up for five years is planned, making it possible to exclude subjects that develop chronic cognitive impairment. Ethical approval was given from the Regional Committees for Medical and Health Research Ethic in Southeastern Norway.

The Oslo Parkinson’s disease Cohort Study recruited normal controls among spouses to participants diagnosed with PD, and from social clubs and among patients in general practice in the Oslo area. The normal controls were without any sign of neurological diseases, all were >40 years of age, had no known parkinsonism or other neurodegenerative disorder. Ethical approval was given from the Regional Committees for Medical and Health Research Ethic in Southeastern Norway.

**European Study Groups**

The Sant Pau Hospital in Barcelona, Spain, included patients with a diagnosis of probable dementia with Lewy Bodies (McKeith 2005 criteria) from January 2006 to September 2015 (n=120). They were referred to the Memory Unit by a primary care physician, a general neurologist or other medical specialties. All patients underwent a complete neuropsychological assessment and a specific neurological evaluation directed at DLB signs and symptoms. All of them underwent structural neuroimaging and blood tests and their treatments were systematically reviewed to exclude other causes for the presence of the diagnostic DLB signs and symptoms. We also retrospectively assessed the established criteria for probable DLB independently of the clinical evaluation by clinicians during the follow up. Subsets of patients underwent functional neuroimaging and/or a lumbar puncture to study the classical Alzheimer disease (AD) biomarkers in cerebrospinal fluid (CSF). On the other hand, healthy subjects recruited in the Memory Unit – Sant Pau Hospital (n = 67) were asymptomatic volunteers with no cognitive complains, normal neuropsychological performance, absence of neurological or psychiatric history, and normal levels of all three AD core biomarkers (total tau, phosphorylated tau and $\beta$-amyloid1-42 proteins) in CSF. The ethics committee from Sant Pau hospital approved the use of all samples recruited from this institution that have been used in this study.

In the Barcelona Alzheimer Treatment & Research Center (ACE)-study DLB patients (n=188) fulfilling the McKeith criteria from 2005 were included and referred to the Memory Clinic by their primary care physician, a consulting neurologist or a psychiatrist. All of the patients received a standardized neurobehavioral exam, including neuropsychological and social work evaluation, as well as appropriate laboratory and neuroimaging studies (brain CT or MRI). All diagnoses were assigned by the clinical team at a daily consensus conference. The final diagnosis is based on the results of the neurological, neuropsychological and social work evaluations. The normal controls (n=288) had normal neuropsychological performance after a comprehensive assessment and were without cognitive complaints. The study was approved by an external ethics committee: “CEIC. Hospital Clinic, Barcelona, Spain”. The genetic collection has a wide informed consent permitting GWAS, exome and/or whole genome sequencing of patients. The informed consent also approves data sharing and data access of external experts. Reference number for Genetic informed consent approval is HC140606. The genetic collection is registered in the Ministry of Health of Spain (ISCIII Collection - C.0000299).

The Rate of Alpha-synuclein in Cerebrospinal Fluid to Differentiate Patients With Alzheimer's Disease From Those With Lewy Body Disease (The AlphaLewyMA) study is a prospective cohort of patients with prodromal and mild dementia. The aim of the study is to find early biomarkers to differentiate DLB from AD. Patients at least 45 years of age diagnosed with DLB and AD were included in this study. Patients who meet both AD and DLB-criteria, who could not participate in a lumbar puncture, or were already diagnosed with a neurological or psychiatric disorder or severely impaired vision or hearing were excluded from this study. The ethics committee "Comité de protection des personnes Est IV" approved the genetic research for the ALphaLewyMA study.

Patients with probable DLB were included from the Amsterdam Dementia Cohort of the VU University Medical Center who visited the memory clinic between January 2004 and July 2014. All patients underwent extensive standardized dementia screening, including medical history, physical and neurological examination, electroencephalography, magnetic resonance imaging and routine laboratory tests and lumbar puncture. Diagnoses were made by consensus in a multidisciplinary team according to the current international diagnostic criteria. An additional dopamine transporter imaging with ^[123I]^FP-CIT single photon emission computed tomography (SPECT) was performed in case of diagnostic uncertainty. The local Medical Ethics Committee has approved a general protocol for biobanking and use of the clinical data for research purposes. The Medical Ethical Review Committee of VU University Medical Center has approved a general protocol for biobanking and using the clinical data for research purposes.

The DLB patients from Malmö were part of a larger cohort which was screened for participation in a RCT study. Thereafter they were included in a follow up program at the Memory clinic. The diagnostic process included CT and laboratory tests for exclusion of treatable disorders, neurological and psychiatric clinical investigations and cognitive testing including the MMSE. ADL functioning was measured with DAD, parkinsonism with UPDRS, fluctuations with Mayo fluctuation scale, quality of life with QoLAD and neuropsychiatric symptoms with NPI. DAT-scan/ CIT-SPECT and lumbar puncture were performed in a subgroup. The clinical diagnoses were made according to the 2005 McKeith DLB consensus criteria and validated by Elisabeth Londos. The study was approved by the Regional ethics committee at Lund University, Sweden.

Patients from Reykjavik, Iceland were clinical cases, assessed at the Memory Clinic, Landspitali University Hospital. They were all evaluated by global cognitive tests including MMSE, Clock Drawing Test and the CERAD ten word memory test (working memory, recall and recognition). Also included in the work- up was MRI and in some cases CSF analysis of AD markers and in a few cases CIT SPECT. Patients with mild cognitive impairment underwent extensive neuropsychological evaluation. Controls were individuals, participating in various research projects at deCODE Genetics in Reykjavik, excluding those that had a diagnosis of a neurodegenerative disorder. Icelandic population approval for the study was obtained from the National Bioethics Committee and the Icelandic Data Protection Authority.

# Supplemental Table 1: DLB and control cohorts

|  | **DLB** | | | **Controls** | | |
| --- | --- | --- | --- | --- | --- | --- |
| **Cohorts** | **N** | **% Women** | **Age** | **N** | **% Women** | **Age** |
| **Cohort 1** |  |  |  |  |  |  |
| AHUS | 16 | 31.2 | 76.5 ±4.7 | 79 | 50.6 | 64.0 ±9.0 |
| COGNORM |  |  |  | 100 | 46.0 | 73.5±6.6 |
| DDI |  |  |  | 111 | 67.5 | 69.3 ±9.2 |
| DemVest | 53 | 49.0 | 81.2 ±6.6 |  |  |  |
| HUNT | 36 | 41.6 | 84.2 ±7.0 | 143 | 55.9 | 78.2 ±9.1 |
| NorCog | 43 | 48.8 | 73.3 ±7.7 |  |  |  |
| Nord Norge |  |  |  | 196 | 41.3 | 67.5 ±5.6 |
| PADR | 5 | 40.0 | 78.4 ±2.6 |  |  |  |
| Trønderbrain | 103 | 39.8 | 82.2 ±10.2 | 682 | 59.2 | 79.4 ±10.0 |
| Amsterdam | 124 | 18.5 | 68.0 ±8.0 |  |  |  |
| Lund | 38 | 28.9 | 80.2 ±5.7 |  |  |  |
| Strasbourg | 60 | 56.7 | 69.5 ±9.2 | 11 | 54.5 | 64.4±6.4 |
| **Total** | **478** | **37.2** | **77.9 ±9.8** | **1322** | **51.7** | **70.5 ±13.5** |
| Norwegian population control |  |  |  | **4875** | 47.9 |  |
| **Total Cohort 1** | **478** | **37.2** | **77.9 ±9.8** | **6197** | **49.6** |  |
|  |  |  |  |  |  |  |
| **Cohort 2** |  |  |  |  |  |  |
| ACE | 181 | 43 | 79.7 ±6.2 | 228 | 57 | 65.4 ± 7.0 |
| Sant Pau | 61 | 64 | 76.3 ±10.0 | 65 | 35 | 69.1 ± 9.6 |
| **Total Cohort 2** | **242** |  |  | **293** |  |  |
| **Total Cohort 3** | **108** |  |  | **75545** |  |  |
| **In total** | **828** |  |  | **82035** |  |  |

# Supplemental Table 2: Top SNP associations with DLB

### **Cohort 1 associations with DLB**

| CHR | BP | SNP | A1 | A2 | MAF | OR | SE | P |
| --- | --- | --- | --- | --- | --- | --- | --- | --- |
| 1 | 154913723 | rs34195153 | G | C | 0.0187 | 5.4349 | 0.3863 | 1.02E-06 |
| 1 | 154988255 | rs71628639 | A | C | 0.0206 | 5.5206 | 0.3214 | 3.06E-09 |
| 1 | 155024309 | rs4845407 | G | C | 0.1224 | 1.9172 | 0.1549 | 2.78E-06 |
| 1 | 155027763 | rs10158328 | A | G | 0.4565 | 1.5985 | 0.1104 | 2.13E-06 |
| 1 | 155028522 | rs11264302 | G | A | 0.4566 | 1.5980 | 0.1103 | 2.11E-06 |
| 1 | 155029253 | rs3765087 | C | A | 0.1229 | 1.9175 | 0.1531 | 2.10E-06 |
| 1 | 155030557 | rs11264303 | A | C | 0.4568 | 1.5967 | 0.1100 | 2.09E-06 |
| 1 | 155033317 | rs11264304 | T | C | 0.265 | 1.6742 | 0.1154 | 6.29E-07 |
| 1 | 155033572 | rs11264305 | A | G | 0.2637 | 1.6750 | 0.1150 | 5.61E-07 |
| 1 | 155108167 | rs12726330 | A | G | 0.0209 | 5.2493 | 0.2918 | 2.33E-10 |
| 1 | 155116151 | rs12742181 | T | C | 0.0208 | 5.2193 | 0.2909 | 2.38E-10 |
| 1 | 155121143 | rs35682329 | G | A | 0.0207 | 5.1996 | 0.2904 | 2.42E-10 |
| 1 | 155135036 | rs35749011 | A | G | 0.0206 | 5.1493 | 0.2890 | 2.51E-10 |
| 1 | 155205378 | rs12752133 | T | C | 0.0198 | 5.5732 | 0.3004 | 1.78E-10 |
| 1 | 155206167 | rs2230288 | T | C | 0.0198 | 5.5744 | 0.3004 | 1.78E-10 |
| 1 | 155359992 | rs71628662 | C | T | 0.0195 | 5.7943 | 0.3084 | 2.09E-10 |
| 1 | 155371119 | rs145330152 | C | A | 0.0197 | 5.7310 | 0.3071 | 2.27E-10 |
| 1 | 155388851 | rs12734374 | T | A | 0.0222 | 5.7668 | 0.3090 | 2.51E-10 |
| 1 | 155820734 | rs34032320 | C | G | 0.0201 | 4.8541 | 0.2951 | 2.32E-09 |
| 1 | 156007988 | rs35603727 | A | G | 0.0225 | 4.8801 | 0.2881 | 8.37E-10 |
| 1 | 156030037 | rs34372695 | T | C | 0.0272 | 3.9960 | 0.2690 | 9.27E-09 |
| 1 | 156072217 | rs71630614 | T | C | 0.0279 | 3.6785 | 0.2698 | 7.23E-08 |
| 1 | 156154860 | rs35643925 | A | G | 0.0234 | 4.3180 | 0.3112 | 1.57E-07 |
| 3 | 153512993 | rs34845539 | T | A | 0.3853 | 0.5954 | 0.1252 | 3.85E-06 |
| 4 | 37212825 | rs78821642 | T | C | 0.0105 | 5.8862 | 0.4321 | 4.73E-06 |
| 4 | 37227478 | rs79067229 | T | C | 0.0108 | 6.1115 | 0.4374 | 3.90E-06 |
| 8 | 96471303 | rs3104921 | A | G | 0.016 | 5.0417 | 0.3931 | 4.43E-06 |
| 11 | 1397268 | rs10832817 | T | C | 0.1165 | 1.9090 | 0.1574 | 4.63E-06 |
| 11 | 1397433 | rs11024347 | G | A | 0.116 | 1.9169 | 0.1574 | 3.97E-06 |
| 11 | 1398019 | rs10832843 | A | G | 0.1158 | 1.9170 | 0.1573 | 3.92E-06 |
| 13 | 95836254 | rs10161985 | T | C | 0.1989 | 1.7471 | 0.1294 | 1.50E-06 |
| 13 | 111125015 | rs7337139 | A | G | 0.0185 | 4.7990 | 0.3418 | 3.09E-07 |
| 19 | 45388500 | rs283811 | G | A | 0.2315 | 1.9194 | 0.1552 | 2.77E-06 |
| 19 | 45390333 | rs283815 | G | A | 0.2291 | 1.9177 | 0.1542 | 2.46E-06 |
| 19 | 45392254 | rs6857 | T | C | 0.16 | 2.3374 | 0.1798 | 1.39E-07 |
| 19 | 45394969 | rs184017 | G | T | 0.2256 | 1.9294 | 0.1543 | 2.03E-06 |
| 19 | 45395714 | rs157581 | C | T | 0.2292 | 1.9285 | 0.1543 | 2.05E-06 |
| 19 | 45396219 | rs157582 | T | C | 0.2225 | 1.9303 | 0.1533 | 1.71E-06 |
| 19 | 45396665 | rs59007384 | T | G | 0.2133 | 1.9647 | 0.1578 | 1.80E-06 |
| 19 | 45411941 | rs429358 | C | T | 0.1432 | 2.6597 | 0.1857 | 4.14E-09 |
| 19 | 45422160 | rs12721051 | G | C | 0.166 | 2.7600 | 0.1894 | 2.21E-09 |
| 19 | 45422846 | rs56131196 | A | G | 0.1689 | 2.7711 | 0.1900 | 2.16E-09 |
| 19 | 45422946 | rs4420638 | G | A | 0.169 | 2.7752 | 0.1900 | 2.08E-09 |
| 19 | 45476504 | rs73558179 | G | T | 0.0609 | 2.7662 | 0.2447 | 3.50E-06 |
| 19 | 45476657 | rs2293755 | G | C | 0.0614 | 2.7651 | 0.2447 | 3.54E-06 |

**CHR:** chromosome; **BP:** base pair location (GrCh37); **SNP:** SNP name; **A1/A2:** minor/major allele; **MAF:** minor allele frequency; **OR:** Odds ratio; **SE:** standard error; **P:** p-value

### **B. **Cohort 2 associations with DLB****

| CHR | BP | SNP | A1 | A2 | MAF | OR | SE | P |
| --- | --- | --- | --- | --- | --- | --- | --- | --- |
| 4 | 155606419 | rs276163 | A | C | 0.4627 | 0.4688 | 0.1653 | 4.56E-06 |
| 4 | 155608695 | Rs276164 | G | A | 0.4625 | 0.4677 | 0.1653 | 4.31E-06 |
| 4 | 155608989 | rs23272 | T | G | 0.4624 | 0.4676 | 0.1653 | 4.28E-06 |
| 11 | 95711914 | rs7104987 | G | A | 0.307 | 2.2441 | 0.1754 | 4.05E-06 |
| 11 | 95713315 | rs685252 | C | T | 0.3069 | 2.2462 | 0.1756 | 4.04E-06 |
| 19 | 45410002 | rs769449 | A | G | 0.1067 | 3.6561 | 0.2614 | 7.09E-07 |
| 19 | 45411941 | rs429358 | C | T | 0.1538 | 3.0208 | 0.2303 | 1.58E-06 |
| 19 | 45415713 | rs10414043 | A | G | 0.1129 | 3.56 | 0.2581 | 8.69E-07 |
| 19 | 45415935 | rs7256200 | T | G | 0.113 | 3.5464 | 0.2578 | 9.06E-07 |

**CHR:** chromosome; **BP:** base pair location (GrCh37); **SNP:** SNP name; **A1/A2:** minor/major allele; **MAF:**  minor allele frequency; **P:** p-value; **OR:** Odds ratio; **SE:** standard error

**C. Stage 1 Meta-analysis associations with DLB**

| CHR | BP | SNP | A1 | A2 | P | OR | SE |
| --- | --- | --- | --- | --- | --- | --- | --- |
| 1 | 155371119 | rs145330152 | C | A | 1.83E-08 | 4.4619 | 0.24714 |
| 1 | 155388851 | rs12734374 | T | A | 4.30E-09 | 4.8913 | 0.251389 |
| 1 | 156007988 | rs35603727 | A | G | 5.88E-09 | 4.3471 | 0.234806 |
| 1 | 156030037 | rs34372695 | T | C | 8.11E-08 | 3.4994 | 0.217134 |
| 1 | 156072217 | rs71630614 | T | C | 4.36E-07 | 3.2716 | 0.21815 |
| 16 | 88564027 | rs968461 | T | C | 9.24E-07 | 1.624 | 0.091893 |
| 16 | 88568831 | rs4782371 | G | T | 5.55E-07 | 1.63 | 0.09076 |
| 16 | 88570007 | rs1353984 | G | A | 3.45E-07 | 1.6507 | 0.091445 |
| 16 | 88570383 | rs868874 | C | T | 3.81E-07 | 1.65 | 0.091702 |
| 16 | 88570480 | rs899732 | G | A | 3.12E-07 | 1.6532 | 0.09138 |
| 16 | 88570675 | rs749681 | C | T | 4.86E-07 | 1.6398 | 0.091407 |
| 16 | 88570816 | rs749679 | G | A | 3.70E-07 | 1.6504 | 0.091652 |
| 16 | 88572056 | rs12926163 | C | T | 1.45E-07 | 1.687 | 0.09248 |
| 16 | 88575439 | rs8045833 | A | G | 2.80E-07 | 1.7266 | 0.098875 |
| 16 | 88578088 | rs36075092 | G | A | 4.60E-07 | 1.6742 | 0.09504 |
| 19 | 45387459 | rs12972156 | G | C | 1.24E-07 | 2.0811 | 0.128897 |
| 19 | 45387596 | rs12972970 | A | G | 1.24E-07 | 2.0806 | 0.128856 |
| 19 | 45388130 | rs34342646 | A | G | 4.14E-08 | 2.1331 | 0.128451 |
| 19 | 45390333 | rs283815 | G | A | 6.16E-09 | 1.9389 | 0.105935 |
| 19 | 45392254 | rs6857 | T | C | 1.52E-10 | 2.2882 | 0.120207 |
| 19 | 45394336 | rs71352238 | C | T | 9.95E-09 | 2.2108 | 0.128723 |
| 19 | 45394969 | rs184017 | G | T | 2.65E-09 | 1.9684 | 0.105811 |
| 19 | 45395619 | rs2075650 | G | A | 2.26E-08 | 2.1664 | 0.128592 |
| 19 | 45395714 | rs157581 | C | T | 1.20E-09 | 1.9975 | 0.105832 |
| 19 | 45395909 | rs34404554 | G | C | 2.29E-08 | 2.1686 | 0.128809 |
| 19 | 45396144 | rs11556505 | T | C | 2.14E-08 | 2.1714 | 0.128757 |
| 19 | 45396219 | rs157582 | T | C | 9.42E-10 | 1.9997 | 0.105321 |
| 19 | 45396665 | rs59007384 | T | G | 4.53E-09 | 1.9818 | 0.108485 |
| 19 | 45406673 | rs10119 | A | G | 3.06E-07 | 1.6687 | 0.093012 |
| 19 | 45410002 | rs769449 | A | G | 1.39E-10 | 2.6165 | 0.139378 |
| 19 | 45411941 | rs429358 | C | T | 2.00E-14 | 2.7965 | 0.125004 |
| 19 | 45415713 | rs10414043 | A | G | 3.25E-10 | 2.5894 | 0.140744 |
| 19 | 45415935 | rs7256200 | T | G | 3.84E-10 | 2.5789 | 0.14073 |
| 19 | 45416741 | rs438811 | T | C | 9.97E-07 | 1.6922 | 0.099993 |
| 19 | 45421254 | rs12721046 | A | G | 4.16E-08 | 2.1646 | 0.130956 |
| 19 | 45422160 | rs12721051 | G | C | 8.63E-11 | 2.3161 | 0.120364 |
| 19 | 45422846 | rs56131196 | A | G | 7.79E-11 | 2.3202 | 0.120328 |
| 19 | 45422946 | rs4420638 | G | A | 7.53E-11 | 2.3217 | 0.120327 |

**CHR:** chromosome; **BP:** base pair location (GrCh37); **SNP:** SNP name; **A1/A2:** minor/major allele; **P:** p-value; **OR:** Odds ratio; **SE:** standard error

# Supplemental Table 3: FUMA prioritized genes from Stage 1 meta-analysis

Prioritization for protein-coding genes by FUMA is based on a combination of positional mapping, expression quantitative trait loci (eQTL) mapping and chromatin interaction mapping. Details of the methods can be found in ^25^.

| **Locus** | **Gene** | **Chr** | **Start** | **End** | **Strand** | **Pos Map** | **eQTL Map** | **CI Map** | **min p-value** |
| --- | --- | --- | --- | --- | --- | --- | --- | --- | --- |
| 1 | HAX1 | 1 | 154244987 | 154248351 | 1 | 0 | 0 | Yes | 2.89E-06 |
| 1 | AQP10 | 1 | 154293566 | 154297801 | 1 | 0 | 0 | Yes | NA |
| 1 | ATP8B2 | 1 | 154298029 | 154323783 | 1 | 0 | 0 | Yes | NA |
| 1 | SHE | 1 | 154442248 | 154474589 | -1 | 0 | 0 | Yes | NA |
| 1 | TDRD10 | 1 | 154474695 | 154520623 | 1 | 0 | 0 | Yes | NA |
| 1 | UBE2Q1 | 1 | 154521053 | 154531504 | -1 | 0 | 0 | Yes | NA |
| 1 | CHRNB2 | 1 | 154540257 | 154552502 | 1 | 0 | 0 | Yes | NA |
| 1 | ADAR | 1 | 154554538 | 154600475 | -1 | 0 | 0 | Yes | NA |
| 1 | KCNN3 | 1 | 154669931 | 154842756 | -1 | 0 | 0 | Yes | NA |
| 1 | PMVK | 1 | 154897210 | 154909467 | -1 | 1 | 0 | No | NA |
| 1 | PBXIP1 | 1 | 154916552 | 154928599 | -1 | 1 | 0 | Yes | NA |
| 1 | PYGO2 | 1 | 154929502 | 154936329 | -1 | 0 | 0 | Yes | NA |
| 1 | SHC1 | 1 | 154934774 | 154946871 | -1 | 0 | 0 | Yes | NA |
| 1 | CKS1B | 1 | 154947129 | 154951725 | 1 | 0 | 0 | Yes | NA |
| 1 | FLAD1 | 1 | 154955814 | 154965587 | 1 | 0 | 0 | Yes | NA |
| 1 | LENEP | 1 | 154966062 | 154966791 | 1 | 0 | 0 | Yes | NA |
| 1 | ZBTB7B | 1 | 154975127 | 154990998 | 1 | 1 | 0 | Yes | NA |
| 1 | DCST2 | 1 | 154990996 | 155006257 | -1 | 1 | 0 | Yes | NA |
| 1 | DCST1 | 1 | 155006300 | 155023406 | 1 | 0 | 0 | Yes | NA |
| 1 | ADAM15 | 1 | 155023042 | 155035252 | 1 | 0 | 0 | Yes | NA |
| 1 | EFNA4 | 1 | 155036207 | 155042029 | 1 | 0 | 0 | Yes | NA |
| 1 | EFNA3 | 1 | 155036224 | 155060014 | 1 | 0 | 0 | Yes | NA |
| 1 | EFNA3 | 1 | 155036224 | 155059283 | 1 | 0 | 0 | Yes | NA |
| 1 | EFNA1 | 1 | 155099936 | 155107333 | 1 | 2 | 0 | Yes | NA |
| 1 | SLC50A1 | 1 | 155107820 | 155111329 | 1 | 3 | 0 | Yes | NA |
| 1 | DPM3 | 1 | 155112367 | 155113071 | -1 | 3 | 0 | Yes | NA |
| 1 | KRTCAP2 | 1 | 155141884 | 155145951 | -1 | 1 | 0 | No | NA |
| 1 | GBA | 1 | 155204243 | 155214490 | -1 | 2 | 4 | No | NA |
| 1 | RUSC1 | 1 | 155290687 | 155300905 | 1 | 0 | 0 | Yes | 4.30E-09 |
| 1 | ASH1L | 1 | 155305059 | 155532598 | -1 | 3 | 0 | No | 4.30E-09 |
| 1 | MSTO1 | 1 | 155579979 | 155718153 | 1 | 0 | 1 | Yes | 4.30E-09 |
| 1 | YY1AP1 | 1 | 155629237 | 155658791 | -1 | 0 | 1 | No | 4.30E-09 |
| 1 | GON4L | 1 | 155719508 | 155829191 | -1 | 1 | 0 | Yes | 4.30E-09 |
| 1 | SYT11 | 1 | 155829300 | 155854990 | 1 | 1 | 0 | Yes | 4.30E-09 |
| 1 | RIT1 | 1 | 155867599 | 155881195 | -1 | 0 | 0 | Yes | 5.88E-09 |
| 1 | KIAA0907 | 1 | 155882834 | 155904191 | -1 | 1 | 0 | Yes | 5.88E-09 |
| 1 | RXFP4 | 1 | 155911480 | 155912625 | 1 | 0 | 0 | Yes | 5.88E-09 |
| 1 | ARHGEF2 | 1 | 155916630 | 155976861 | -1 | 0 | 0 | Yes | NA |
| 1 | SSR2 | 1 | 155978839 | 155990750 | -1 | 0 | 0 | Yes | NA |
| 1 | UBQLN4 | 1 | 156005092 | 156023585 | -1 | 2 | 0 | Yes | 5.88E-09 |
| 1 | LAMTOR2 | 1 | 156024543 | 156028301 | 1 | 1 | 0 | Yes | 8.11E-08 |
| 1 | RAB25 | 1 | 156030951 | 156040295 | 1 | 1 | 0 | Yes | 8.11E-08 |
| 1 | LMNA | 1 | 156052364 | 156109880 | 1 | 1 | 0 | No | 4.36E-07 |
| 1 | SEMA4A | 1 | 156117157 | 156147543 | 1 | 1 | 1 | No | 2.89E-06 |
| 1 | SLC25A44 | 1 | 156163880 | 156182587 | 1 | 1 | 0 | Yes | 2.89E-06 |
| 1 | PMF1-BGLAP | 1 | 156182773 | 156213123 | 1 | 0 | 0 | Yes | 2.89E-06 |
| 1 | PMF1 | 1 | 156182784 | 156212874 | 1 | 0 | 0 | Yes | 2.89E-06 |
| 1 | BGLAP | 1 | 156211753 | 156213112 | 1 | 0 | 0 | Yes | 2.89E-06 |
| 1 | PAQR6 | 1 | 156213206 | 156217881 | -1 | 0 | 0 | Yes | 2.89E-06 |
| 1 | SMG5 | 1 | 156219015 | 156252620 | -1 | 0 | 0 | Yes | 2.89E-06 |
| 1 | TMEM79 | 1 | 156252726 | 156262976 | 1 | 0 | 0 | Yes | 2.89E-06 |
| 1 | C1orf85 | 1 | 156259880 | 156265463 | -1 | 0 | 0 | Yes | 2.89E-06 |
| 1 | VHLL | 1 | 156268415 | 156269428 | -1 | 0 | 0 | Yes | 2.89E-06 |
| 1 | TSACC | 1 | 156307105 | 156316786 | 1 | 0 | 0 | Yes | 2.89E-06 |
| 2 | ZFPM1 | 16 | 88519725 | 88603424 | 1 | 27 | 0 | No | 1.45E-07 |
| 3 | ZNF225 | 19 | 44616334 | 44637027 | 1 | 0 | 0 | Yes | 2.00E-14 |
| 3 | PVR | 19 | 45147098 | 45166850 | 1 | 0 | 0 | Yes | 2.00E-14 |
| 3 | PVRL2 | 19 | 45349432 | 45392485 | 1 | 16 | 1 | No | 1.52E-10 |
| 3 | OMM40 | 19 | 45393826 | 45406946 | 1 | 23 | 2 | No | 2.00E-14 |
| 3 | APOE | 19 | 45409011 | 45412650 | 1 | 11 | 0 | No | 2.00E-14 |
| 3 | APOC1 | 19 | 45417504 | 45422606 | 1 | 16 | 2 | No | 2.00E-14 |
| 3 | APOC4-APOC2 | 19 | 45445495 | 45452822 | 1 | 0 | 0 | Yes | 2.00E-14 |
| 3 | APOC4 | 19 | 45445495 | 45452820 | 1 | 0 | 0 | Yes | 2.00E-14 |
| 3 | APOC2 | 19 | 45449243 | 45452822 | 1 | 0 | 0 | Yes | 2.00E-14 |
| 3 | CLPTM1 | 19 | 45457842 | 45496599 | 1 | 0 | 0 | Yes | 2.00E-14 |

**Locus:** Genomic locus number; **Gene:** Prioritized gene; **Chr:** chromosome; **Start:** Starting position of the gene; **End:** Ending position of the gene; **Strand:** Strand of the gene (forward/reverse); **PosMap:** genes mapped by positional mapping (maximum distance 10kb); **eQTLMap:** genes mapped by eQTLs (all tissue types, FDR<0.05); **CIMap:** genes mapped by 3-D Chromatin Interaction mapping (all tissues); **min p-value:** The minimum GWAS p-value of mapped SNPs (if available)

# Supplemental Figure 1. Cohort 1 Principal Component Analysis (PCA) plots

### ****A. Cohort 1 without population controls****


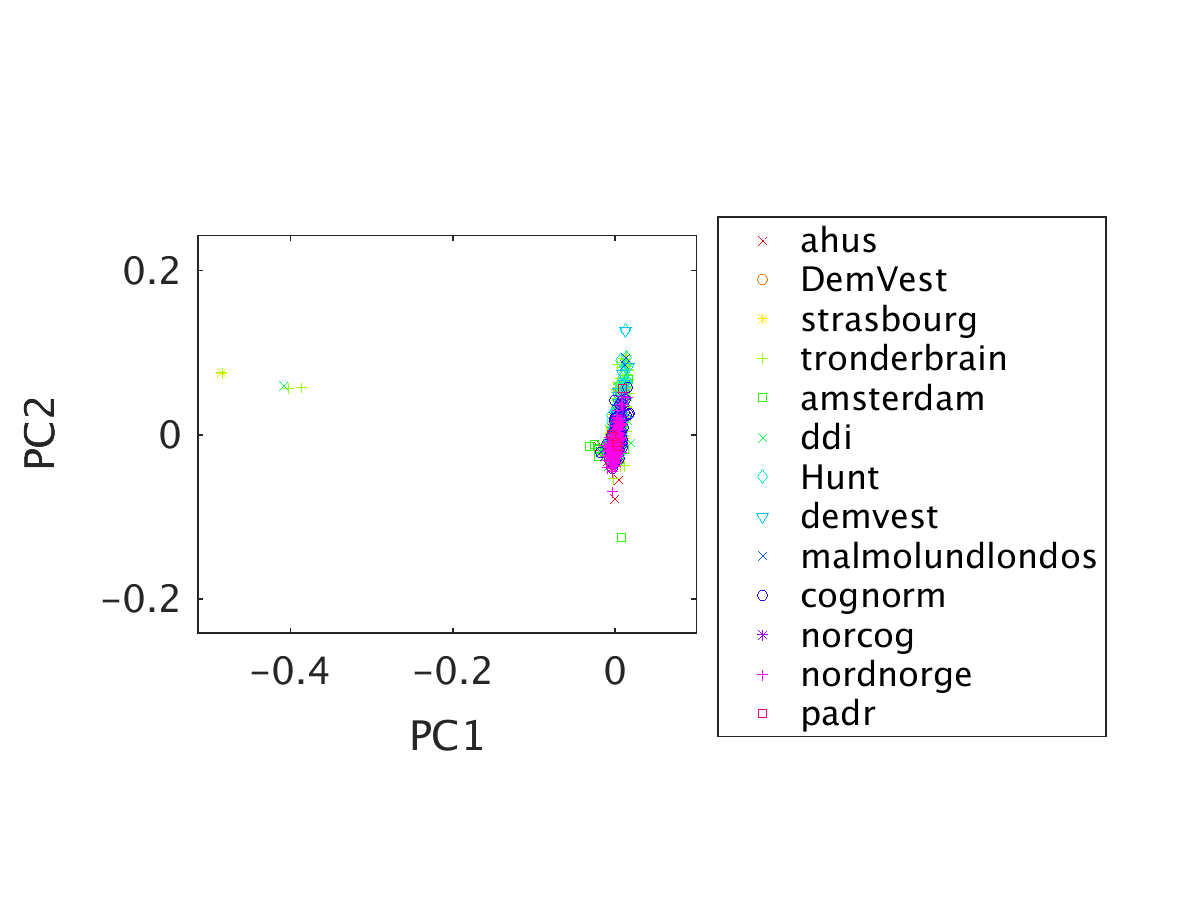


Principal component analysis for Cohort 1 without blood donor population controls: AHUS, DemVest, Strasbourg, TrønderBrain, Amsterdam, DDI, HUNT, DemVest, Malmo/Lund (not available), COGNORM, NorCog, Nord Norge and PADR, a summary of the cohorts is given in S. Table 1 and detailed description is given in S.Text 1.

### ****B. Cohort 1 with population controls****


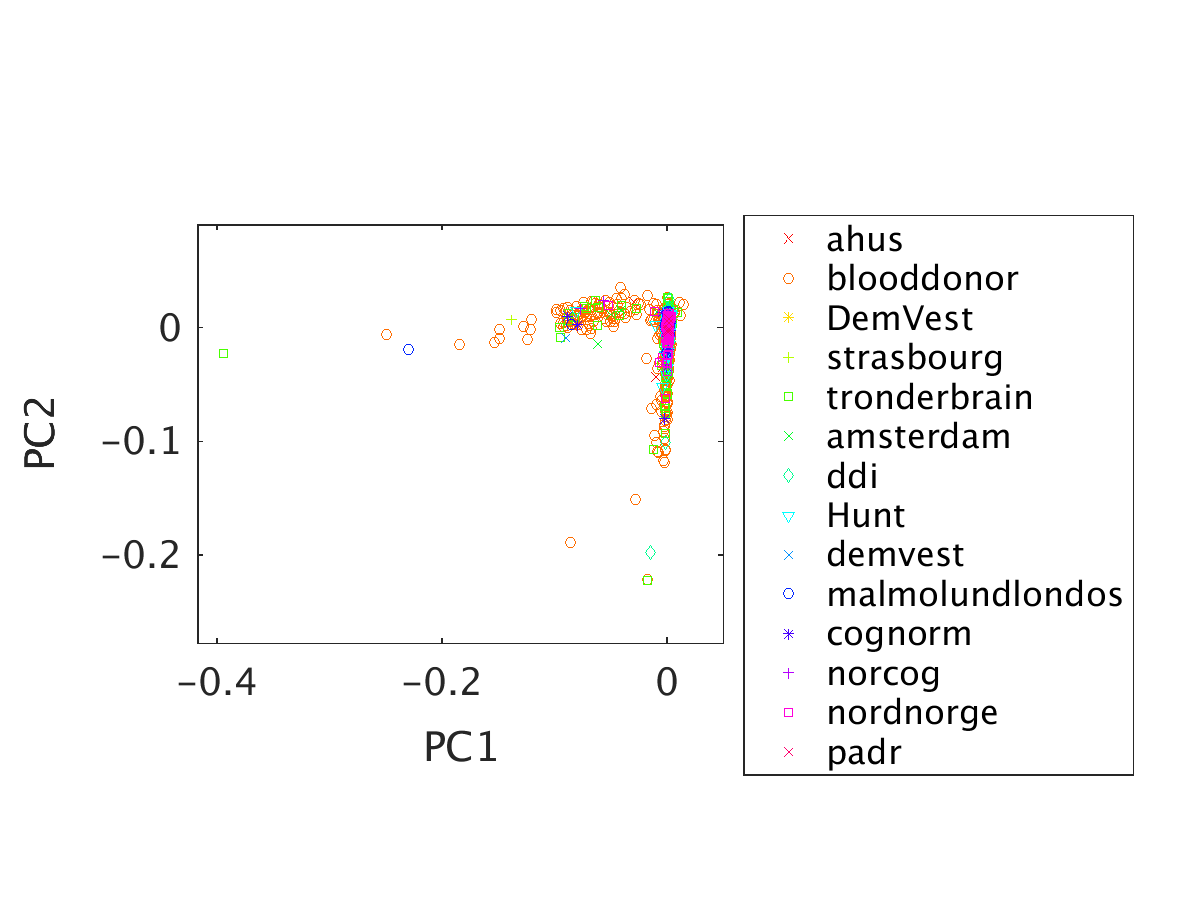


Principal component analysis for Cohort 1 including blood donor population controls (blooddonor) as control for population stratification.

# Supplemental Figures 2: Stage 1 Quantile-quantile (Q-Q) plots

### ****A. Q-Q Cohort 1 without population controls****

Before Genomic Correction (Lambda = 1.003)

**
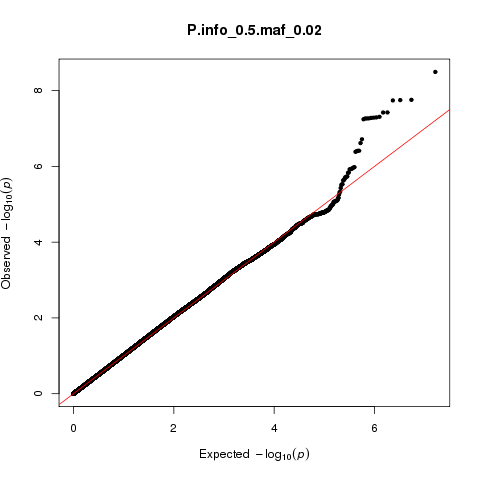
**

### ****B. Q-Q Cohort 1 with population controls****

Before Genomic Correction (Lambda = 1.244)

**
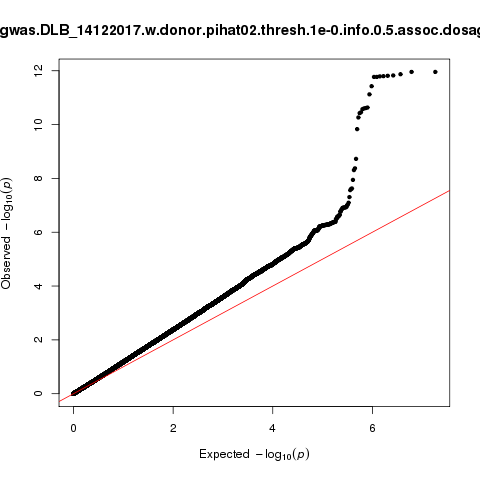
**

After Genomic Correction (Lambda = 1.000)

**
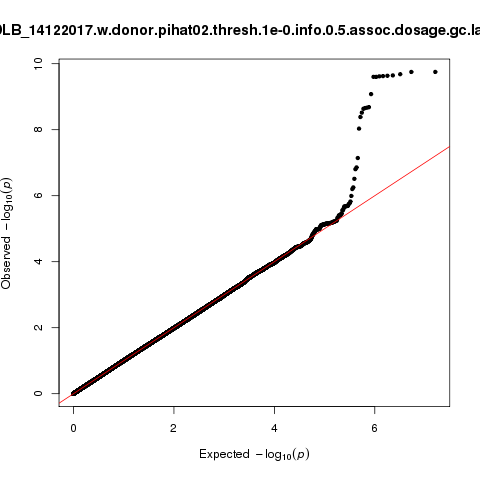
**

Lambda = 1.000

**C. Q-Q Cohort 2**

Before Genomic Correction (Lambda = 1.031)

**
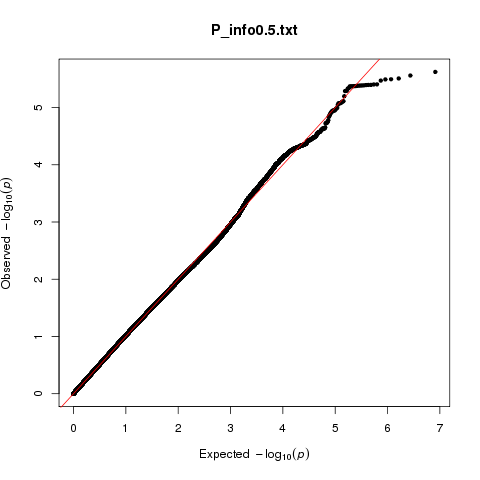
**

### ****D. Meta-analysis Cohort 1 and 2****

Before Genomic Correction (Lambda = 0.865)

**
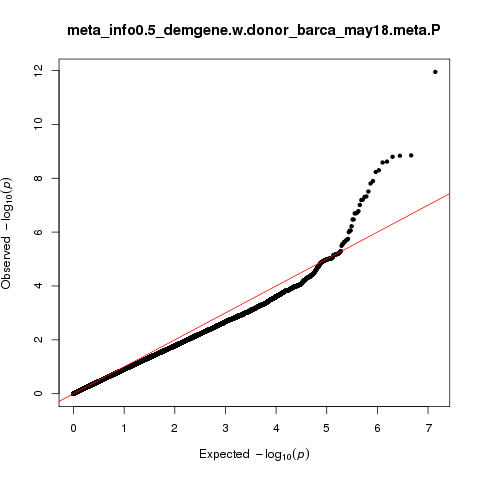
**

After Genomic Correction (Lambda = 1.000)

**
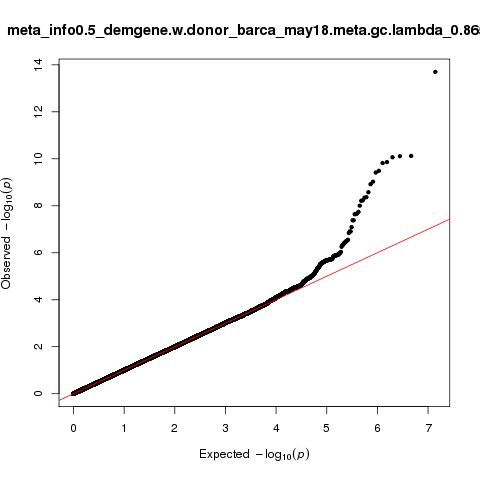
**

#
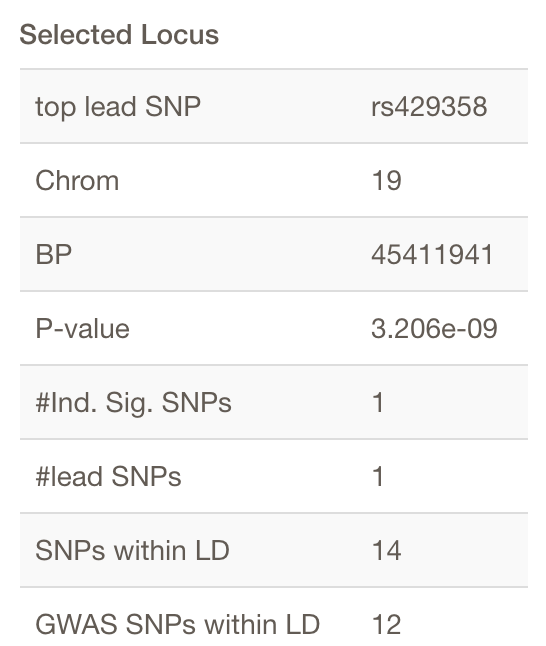
Supplemental Figures 3: Regional locus association plot of Cohort 1 without population controls

### ****
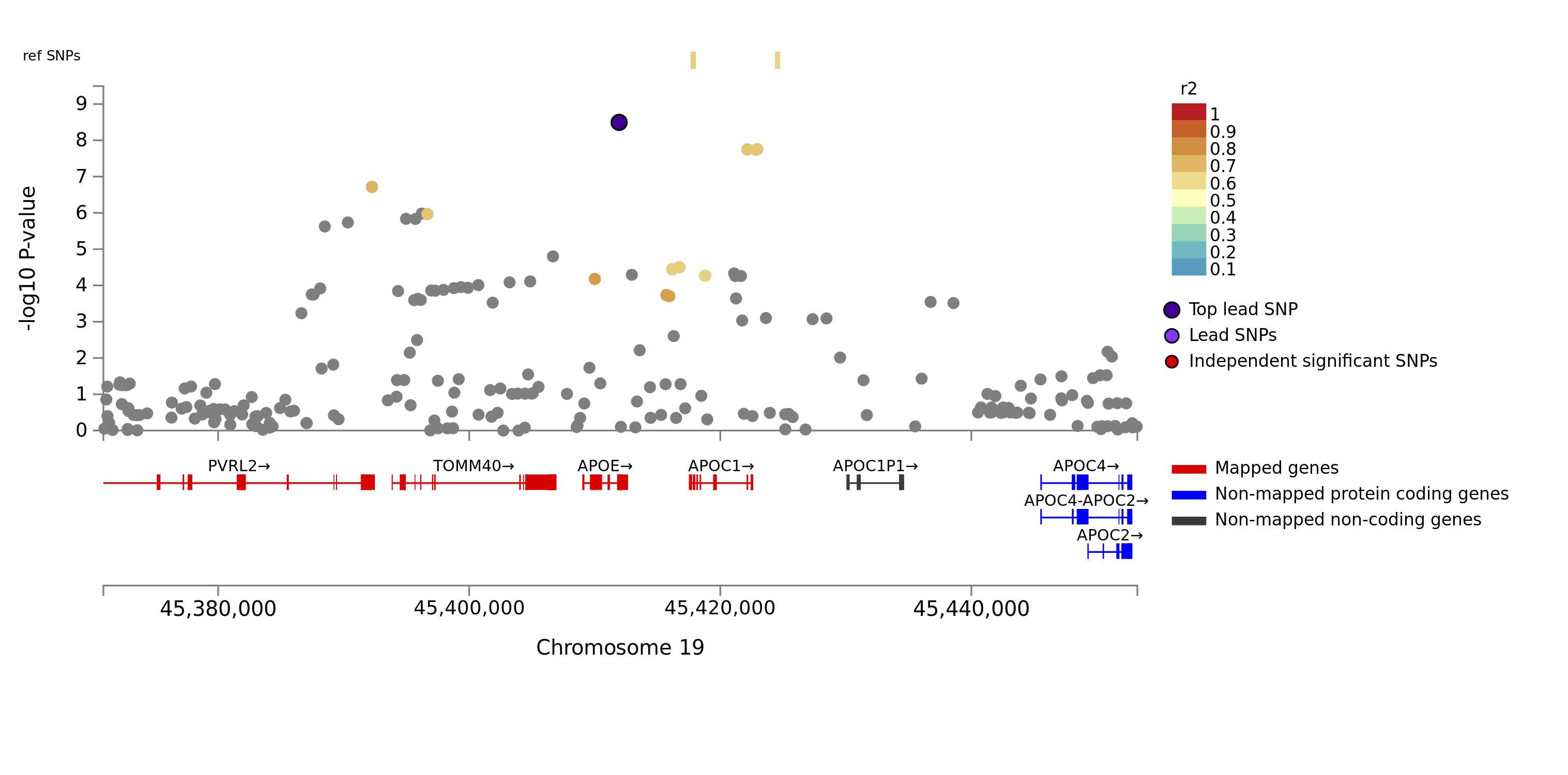
A. APOEe4 locus chromosome 19****

### ****
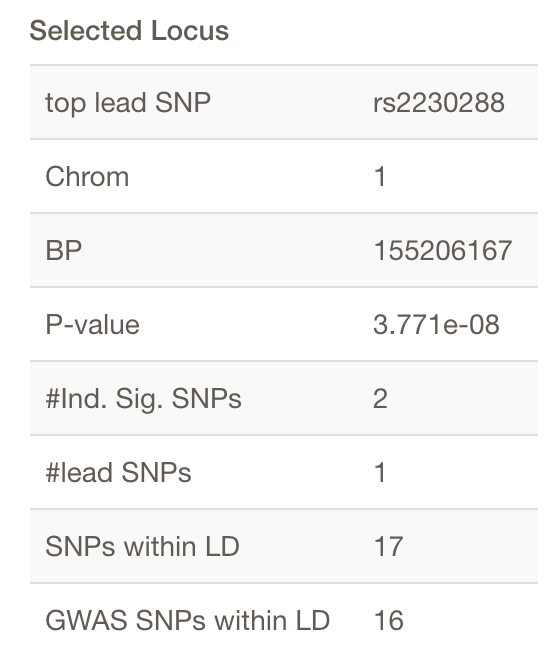
GBA locus chromosome 1****

**
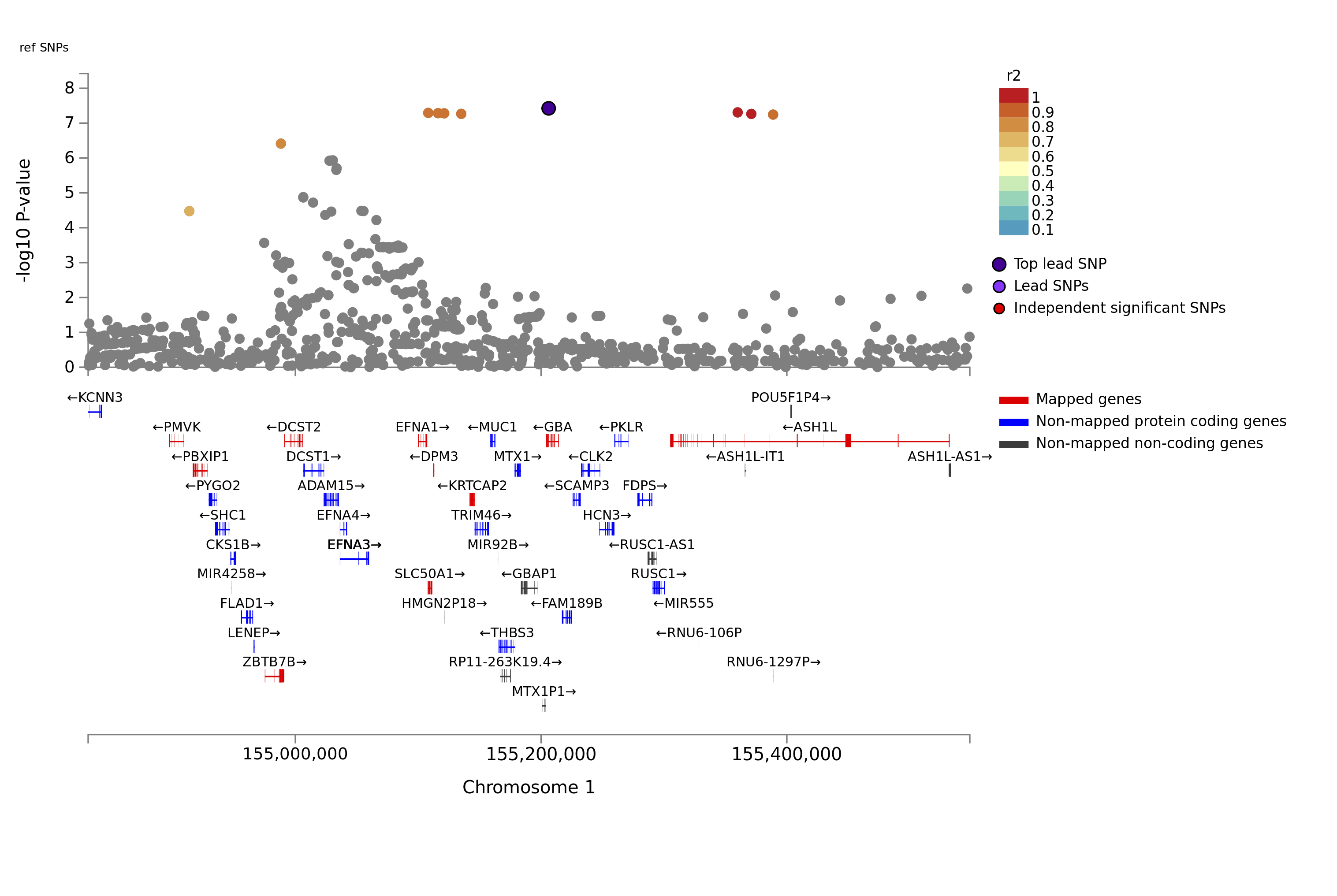
**

# Supplemental Figure 4. Statistical Power of DLB GWAS

### ****Genomic Risk Ratio GRR=1.5****

Statistical power of the study as a function of number cases included in the study. Calculation performed by Genetic Association Study Power Calculator (<http://csg.sph.umich.edu/abecasis/cats/gas_power_calculator>) with 720 cases and 6490 controls at significance level p = 5 x 10^-6^. DLB prevalence is estimated at p=0.0023 and the threshold for genotype relative risk is set at GRR=1.5. Statistical power is estimated at Power=0.937, 0.823, 0.395 and 0.085 for minor allele frequency MAF=0.5, 0.2, 0.1 and 0.05, respectively. Black vertical line indicates the number of cases included in Stage 1 in this study (n=720).

### ****Genomic Risk Ratio GRR=1.2****

Statistical power of the study as a function of number cases included in the study. Calculation performed by Genetic Association Study Power Calculator (<http://csg.sph.umich.edu/abecasis/cats/gas_power_calculator>) with 720 cases and 6490 controls at significance level p = 5 x 10^-6^. DLB prevalence is estimated at p=0.0023 and the threshold for genotype relative risk is set at GRR=1.2. Statistical power is estimated at Power=0.095, 0.026, 0.005 and 0,001 for minor allele frequency MAF=0.5, 0.2, 0.1 and 0.05, respectively. Black vertical line indicates the number of cases included in Stage 1 in this study (n=720).

# Supplemental Figures 5: Regional locus association plots of Meta-Analysis Stage 1

###
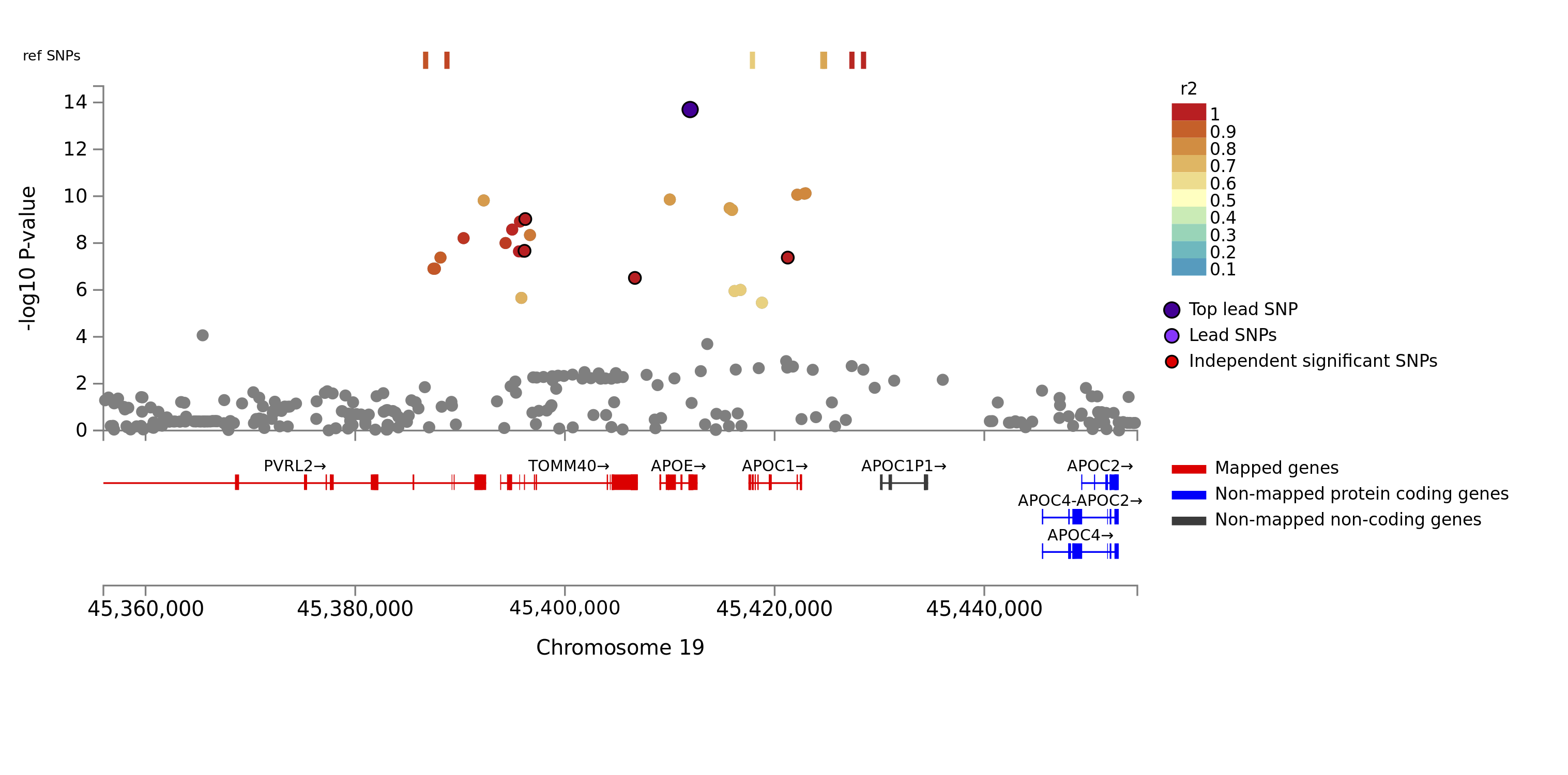

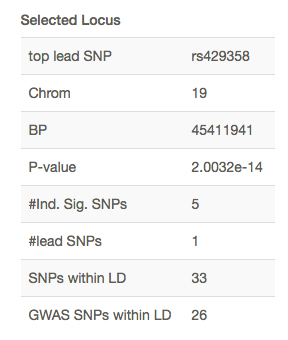
A****. APOEe4 locus chromosome 19****

### **
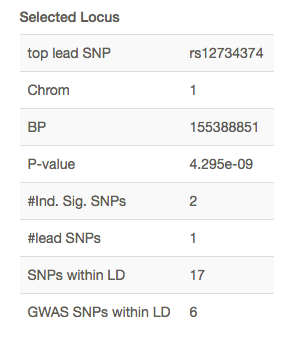
B. **ASH1L/GBA locus chromosome 1****


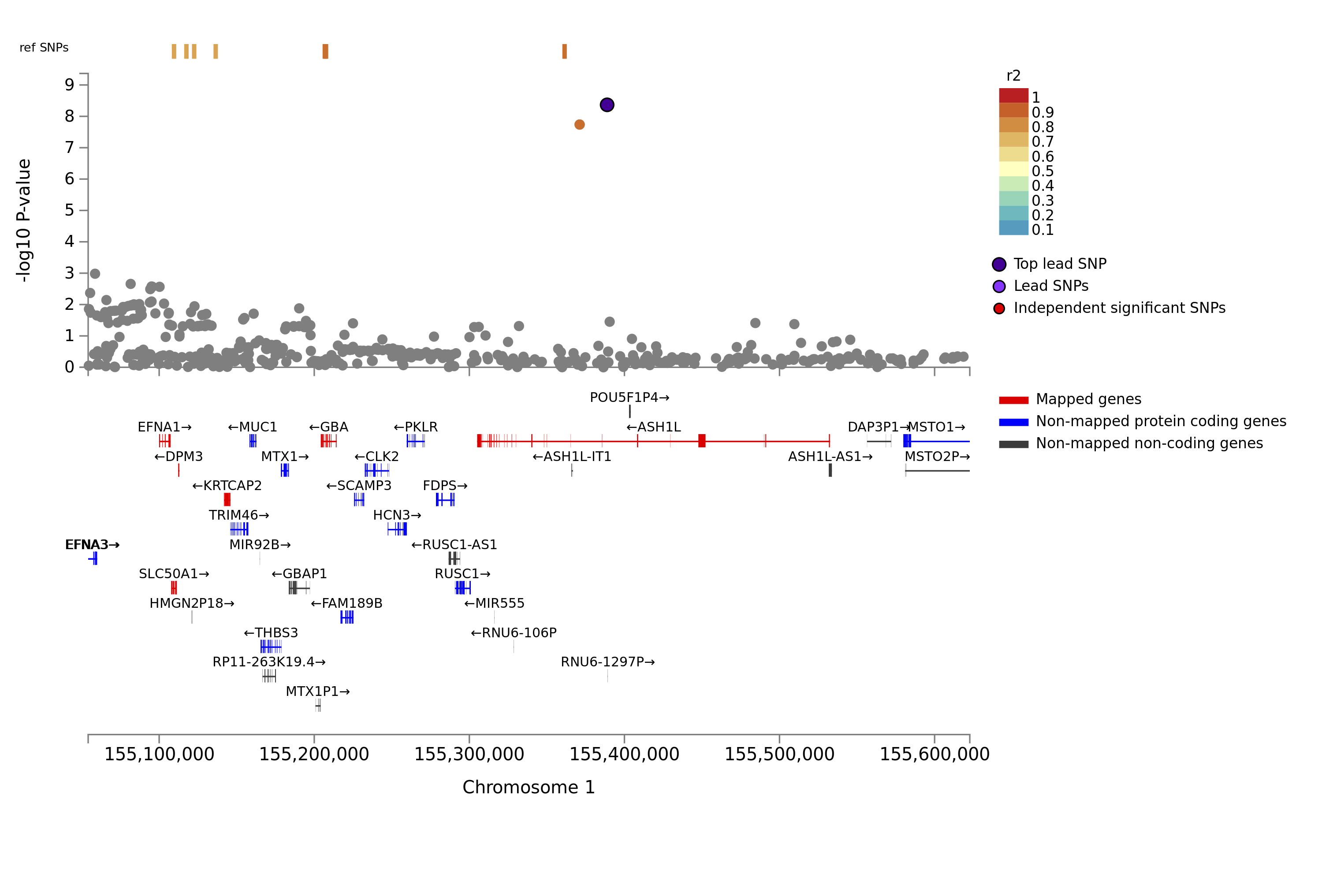


### **
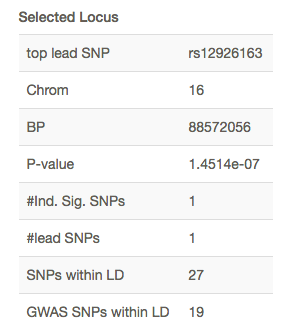
**ZFPM1 locus chromosome 16****

**
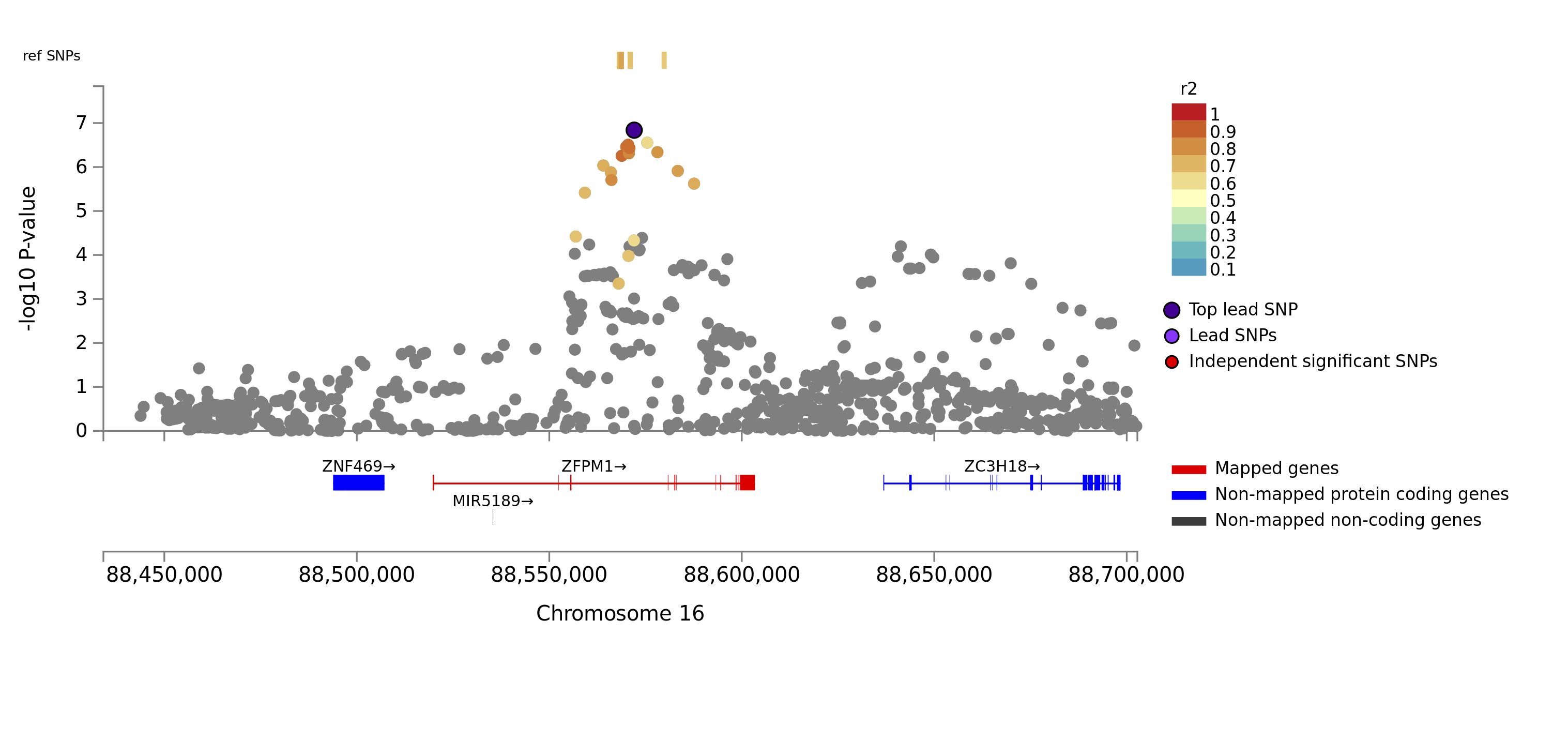
**

### ****SNCA locus chromosome 4****

### ****SCARB2 locus chromosome 4****

### ****MAPT locus chromosome 17****

### ****CNTN1 locus chromosome 12****

# Supplemental Figure 6: FUMA GWAS Summary


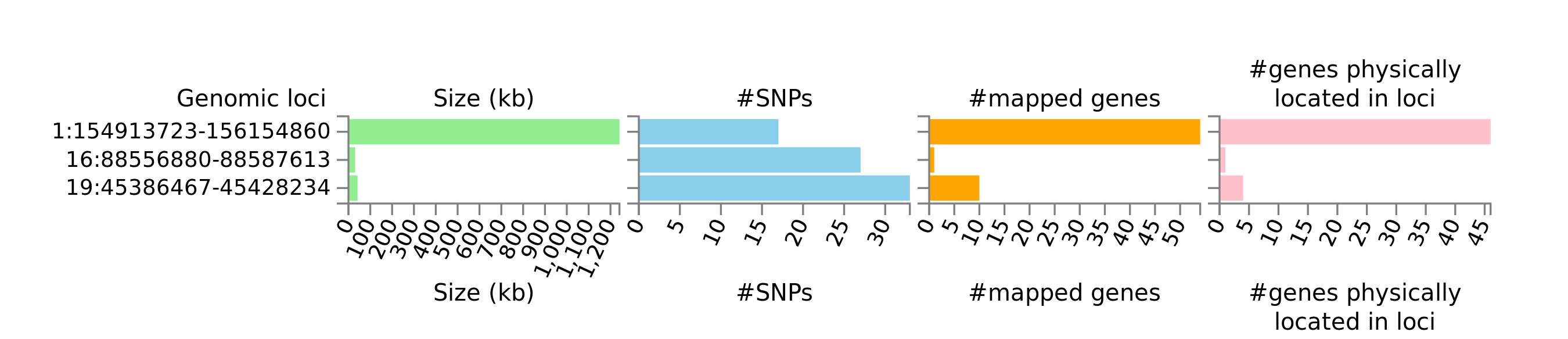


To define genomic loci of interest to the trait based on provided GWAS summary statistics, pre-calculated LD structure based on 1000G reference population EUR is used. Independent suggestive SNPs with a *P*-value < 1e-6 and independent from each other at *r*^2^ < 0.6 are identified. For each independent significant SNP, all known SNPs that have *r*^2^ ≥ 0.6 with one of the independent significant SNPs are included for further annotation (candidate SNPs). Based on the identified independent suggestive SNPs, independent lead SNPs are defined if they are independent from each other at *r*^2^ < 0.1. Additionally, if LD blocks of independent suggestive SNPs are closely located to each other (< 250 kb based on the most right and left SNPs from each LD block), they are merged into one genomic locus. Gene annotation is based on Ensembl genes (build 85). Details of methods are in ^25^. 51 genes are physically located in loci, 65 prioritized genes are mapped by FUMA, presented in Supplementary Table 4.

# Supplemental Figure 7: Gene-based Manhattan plots


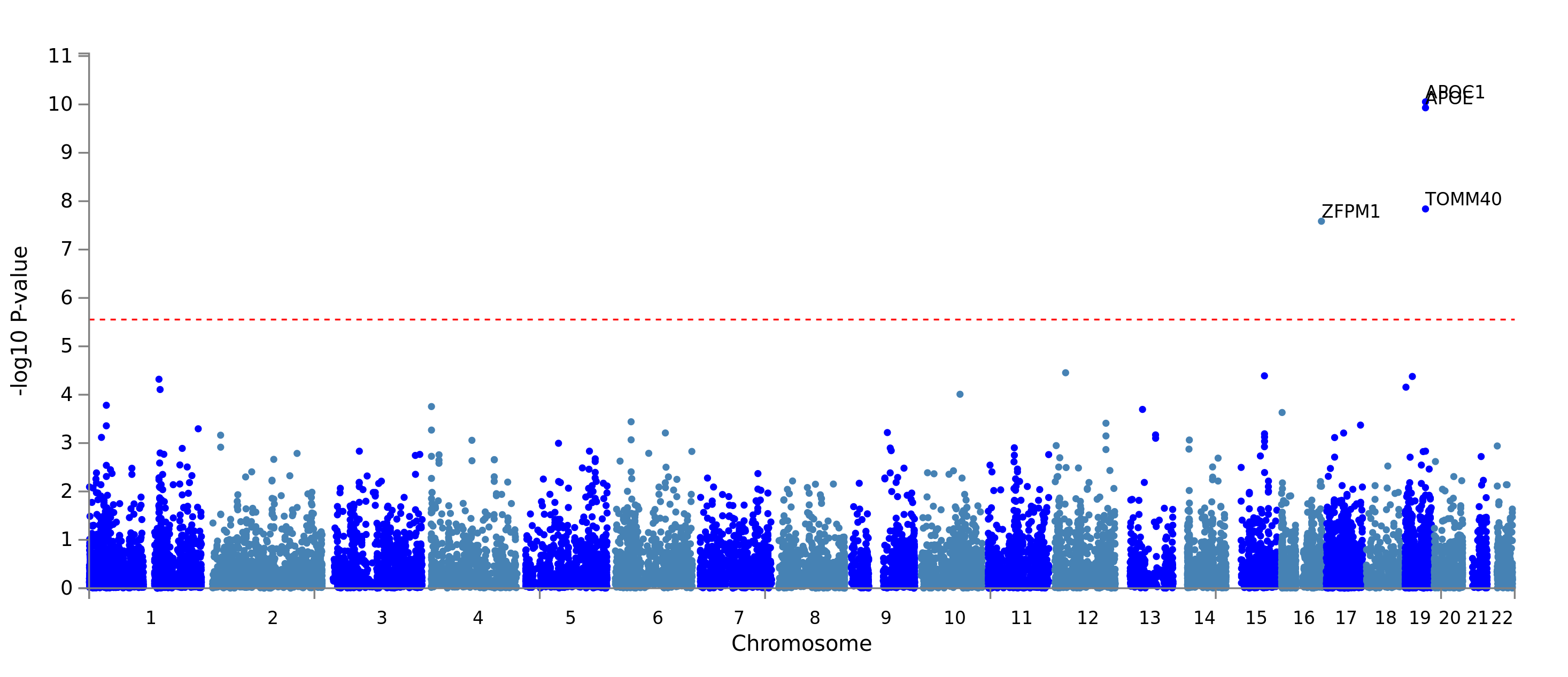


FUMA compute gene-based P-values using the MAGMA tool ^26^. The gene-based P-value is computed for protein-coding genes by mapping SNPs to genes if SNPs are located within the genes. This model first projects the SNP matrix for a gene onto its principal components (PC), pruning away PCs with very small eigenvalues, and then uses those PCs as predictors for the phenotype in the linear regression model. Input SNPs were mapped to 17869 protein coding genes. Genome wide significance (red dashed line in the plot) was defined at P = 0.05/17869 = 2.798e-6. Variants of four genes were identified at genome-wide significance: *APOE, APOC1, TOMM40, ZFPM1*.

# Supplemental Figure 8: MAGMA Tissue Expression Analysis


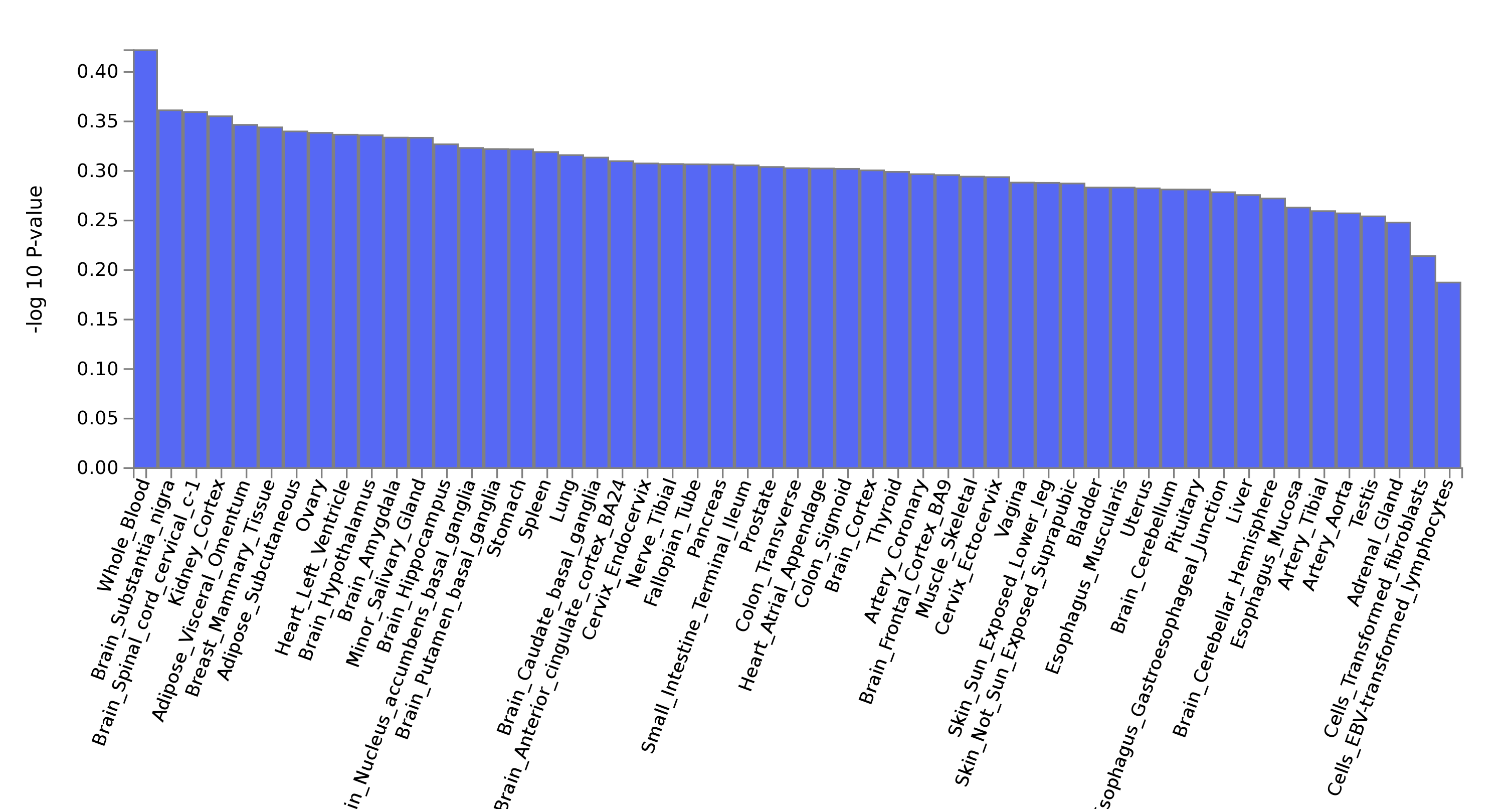


FUMA GWAS MAGMA gene-property analysis is performed for gene expression per tissue based on GTEx RNA-seq data. MAGMA was performed for average expression of 53 tissue types using the full distribution of SNP p-values. In our DLB study, whole blood, substantia nigra and spinal cord cervical c.1 were the most strongly expressed compared to other tissues, but none passed the Bonferroni corrected significance threshold for 53 tests.

# Supplemental Figure 9: Circos plots of chromatin interactions and eQTLs

### ****Chromosome 1****


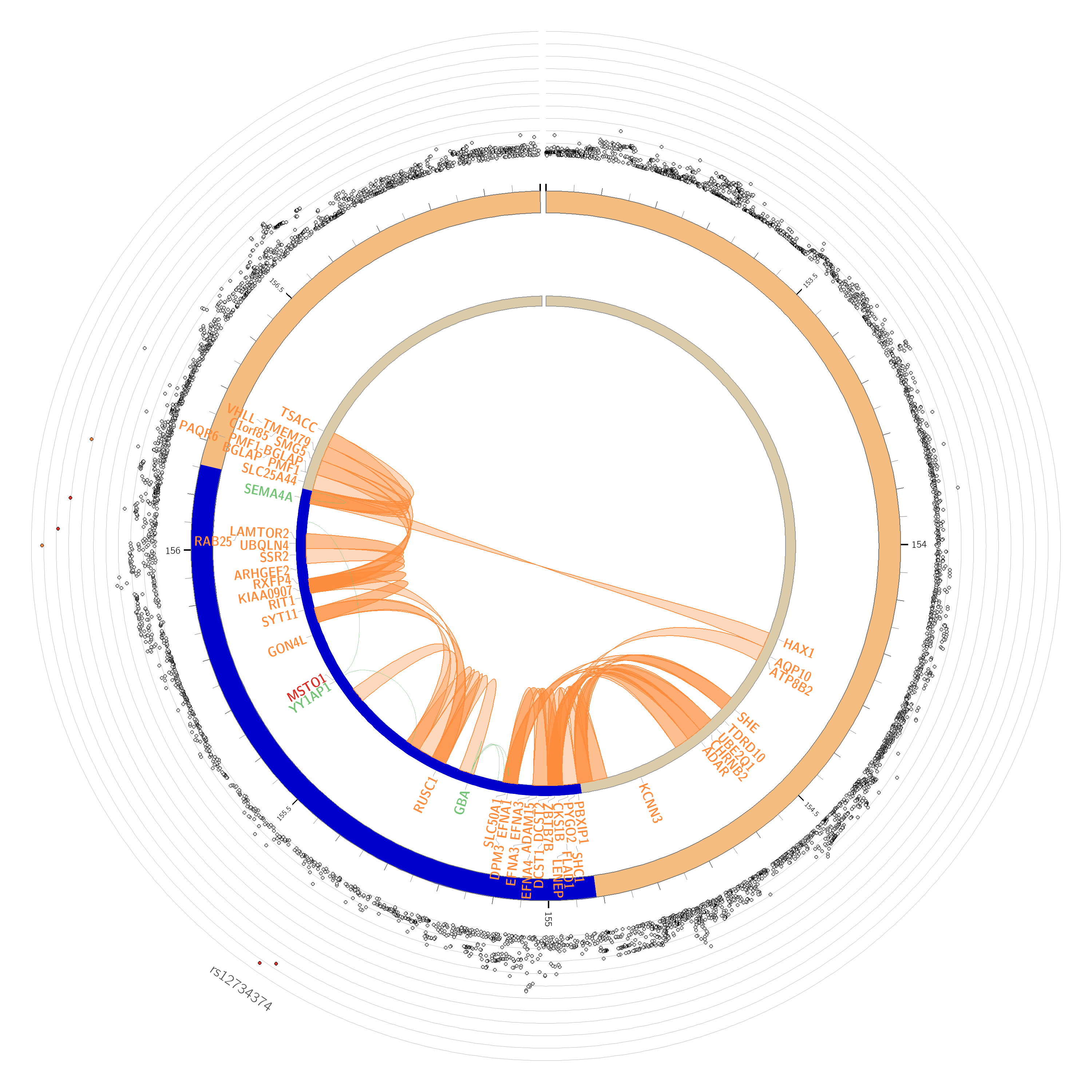


Circos plots showing manhattan plots (outer rings) and mapped genes. Genomic risk loci are highlighted in blue. If the gene is mapped only by chromatin interactions or only by eQTLs, it is colored orange or green, respectively. When the gene is mapped by both, it is colored red.

### ****Chromosome 16****


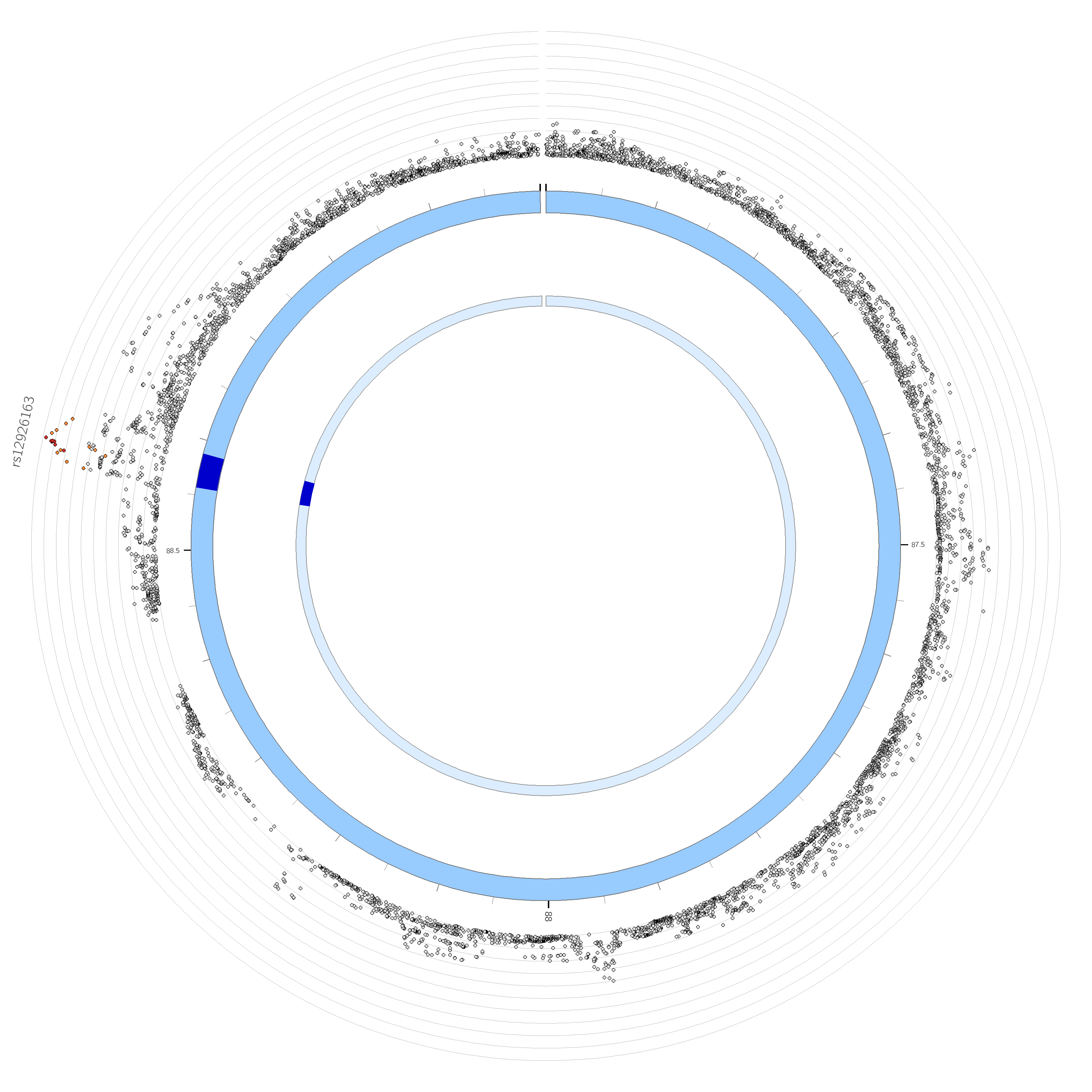


Circos plots showing Manhattan plots (outer rings) and mapped genes. Genomic risk loci are highlighted in blue. If the gene is mapped only by chromatin interactions or only by eQTLs, it is colored orange or green, respectively. When the gene is mapped by both, it is colored red.

### ****Chromosome 19****


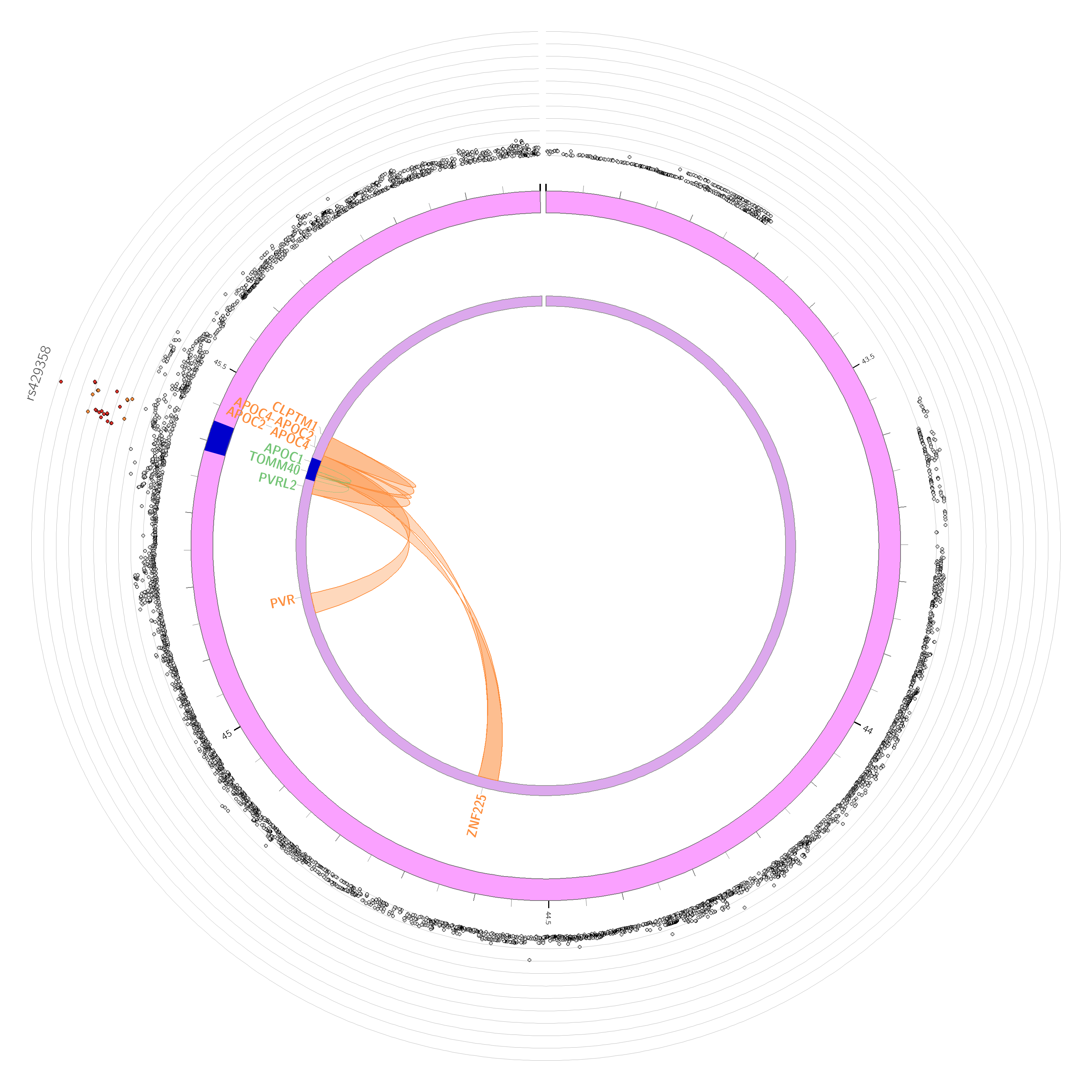
 Circos plots showing Manhattan plots (outer rings) and mapped genes. Genomic risk loci are highlighted in blue. If the gene is mapped only by chromatin interactions or only by eQTLs, it is colored orange or green, respectively. When the gene is mapped by both, it is colored red.

# Supplemental Figure 10: Gene expression heatmap

### ****A. Average expression per label of 53 specific tissue types****

**
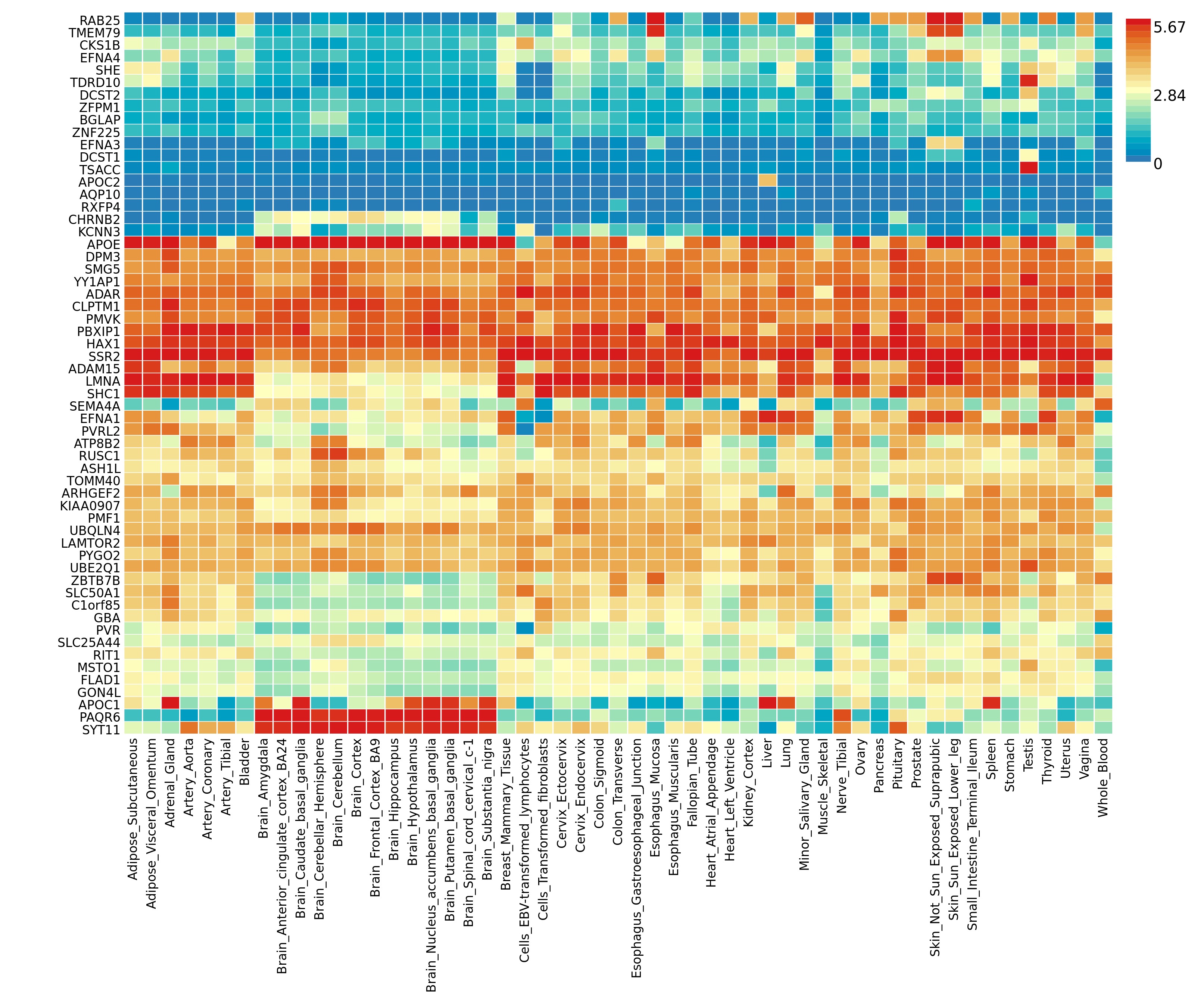
**

Specific expression patterns of 53 tissue types were obtained from GTEx v6 RNA-seq data. Normalized average gene expression levels (RPKM, Read Per Kilobase per Million) of each FUMA prioritized gene are visualized as a heatmap after following RPKM log2 transformation. Darker red means higher expression of that gene, compared to darker blue color. Genes are ordered by clustering of their expression levels.

### ****B. Normalized gene expression heatmap of 53 specific tissue types****

**
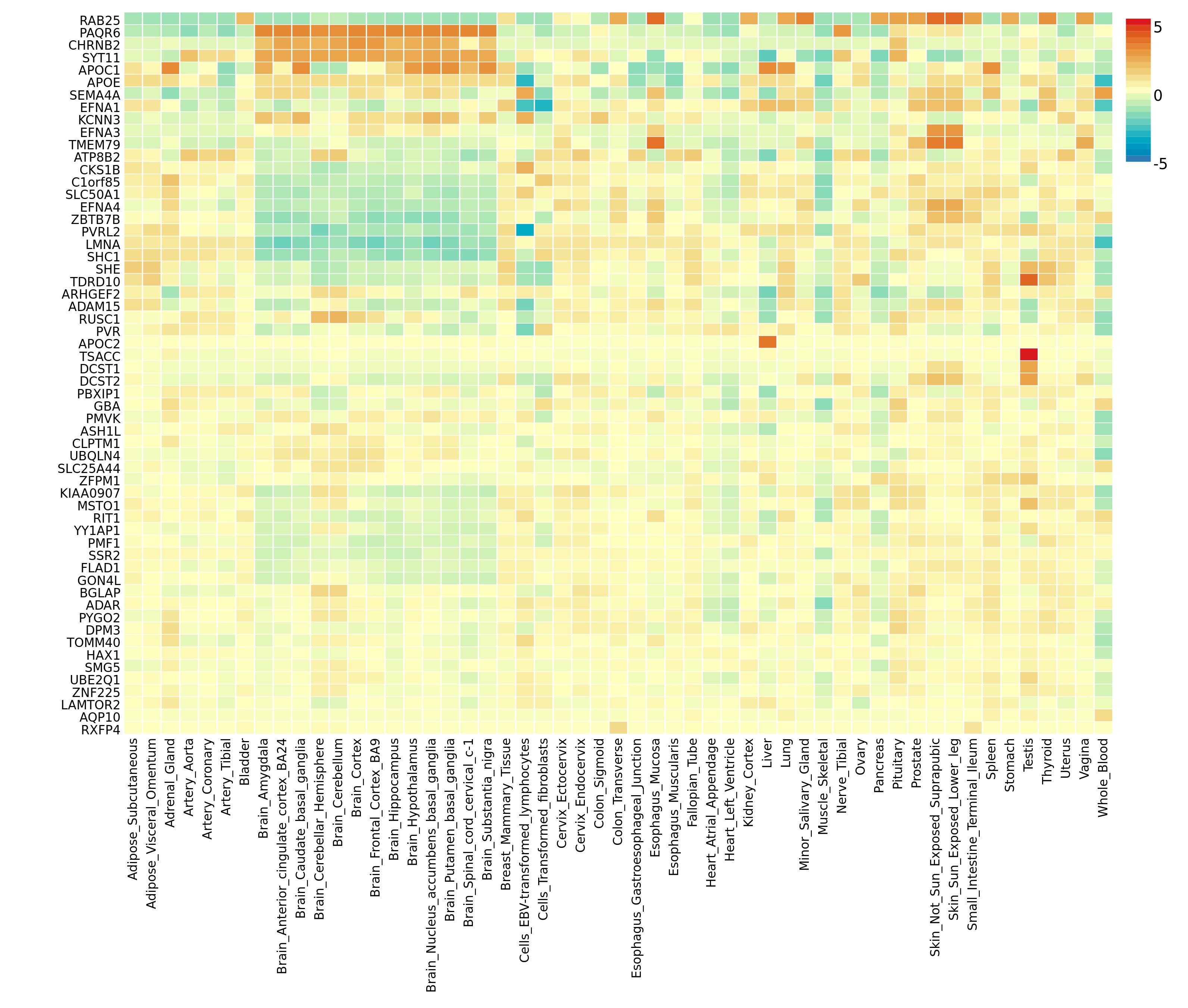
**

Average value of the relative expression value (zero mean normalization of log2 transformed expression). Darker red means higher relative expression of that gene in label X compared to a darker blue color in the same label. Genes are ordered by clustering of their normalized expression levels.

# Supplemental Figure 11: Differentially expressed genes across 53 specific tissue types


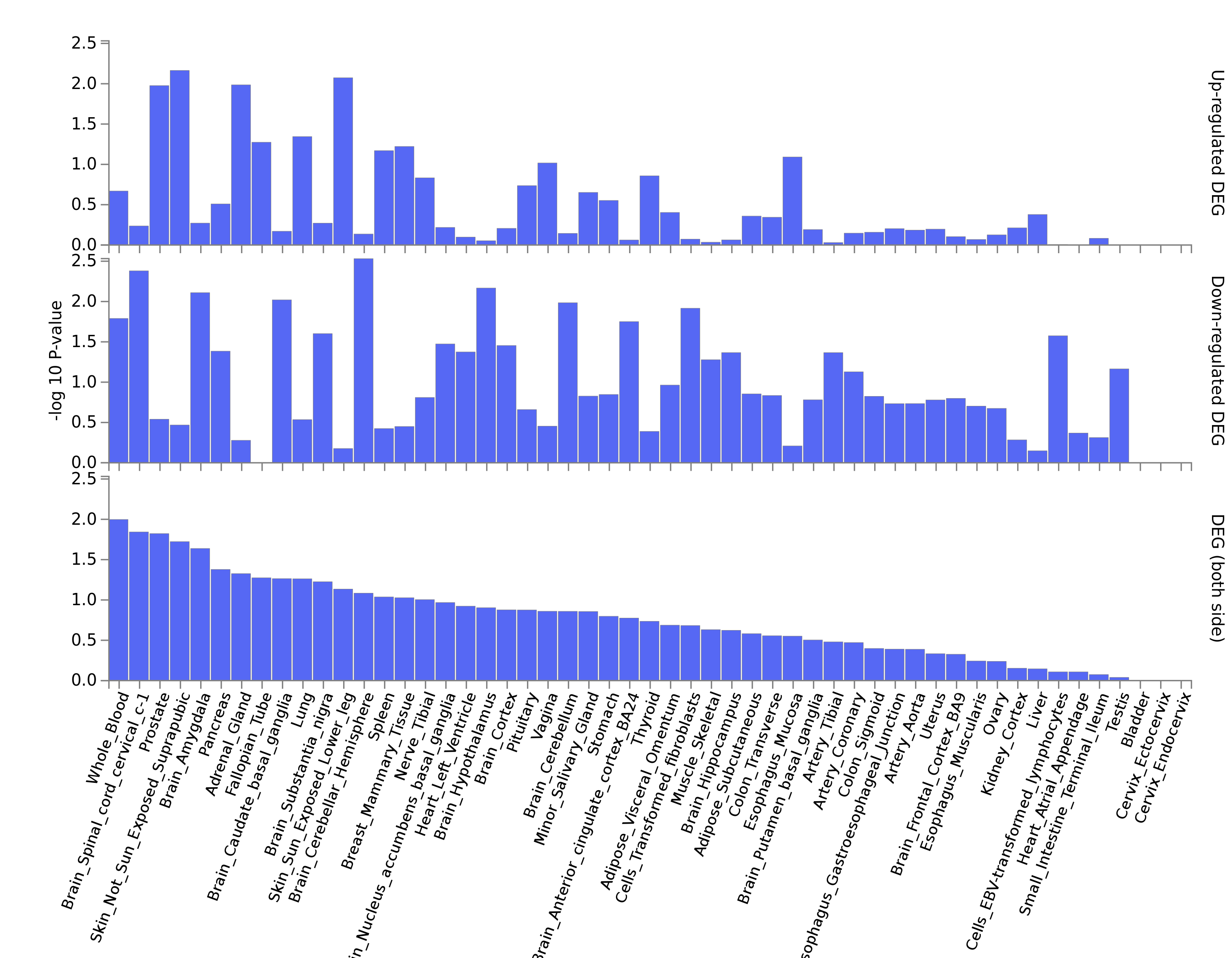


Beside the single gene level analyses, we identify tissue specificity of prioritized genes by looking at overrepresentation in sets of differentially expressed genes (DEG). To obtain DEG for each of 53 specific tissue type based on GTEx v6 RNA-seq, the normalized expression (zero mean of log2(RPKM+1)) is used. Student’s *t*-tests are performed per gene per tissue against all other tissues. After Bonferroni correction, genes with corrected *P*-value < 0.05 and absolute log fold change ≥ 0.58 are defined as a DEG set in a given tissue, i.e., for these genes expression in the given tissue had the largest discrepancy with expression in all other tissues. We found that Brain_Spinal_cord_cervical_c-1 and Brain_Amygdala being in the top five specific tissue, however, they are at norminal significance (Bonferroni threshold for significant p=0.05/53=0.0009 or –log10(p)=3.02).

**References:**

1 Steinberg, S. *et al.* Loss-of-function variants in ABCA7 confer risk of Alzheimer's disease. *Nature genetics* (2015).

2 Aarsland, D. *MULTI-CENTRE COHORT-STUDIES IN LEWY-BODY DEMENTIA*, <<http://www.neurodegenerationresearch.eu/wp-content/uploads/2015/10/JPND-Report-Aarsland.pdf>> (2015).

3 Selnes, P. *et al.* Diffusion tensor imaging surpasses cerebrospinal fluid as predictor of cognitive decline and medial temporal lobe atrophy in subjective cognitive impairment and mild cognitive impairment. *Journal of Alzheimer's Disease* **33**, 723-736 (2013).

4 Aarsland, D. *et al.* Frequency and case identification of dementia with Lewy bodies using the revised consensus criteria. *Dement Geriatr Cogn Disord* **26**, 445-452 (2008).

5 Nåvik, M., Engedal, K. & Ulstein, I. Register til nytte eller besvær, hva vet vi etter fem år? Etablering av et kvalitets-og forskningsregister for hukommelsesklinikker i spesialisthelsetjenesten. *Nordisk tidsskrift for helseforskning* **9**, 92-97 (2014).

6 Bergh, S. *et al.* Cohort Profile: The Health and Memory Study (HMS): a dementia cohort linked to the HUNT study in Norway. *International journal of epidemiology*, dyu007 (2014).

7 Sando, S. B. *et al.* APOE ε4 lowers age at onset and is a high risk factor for Alzheimer's disease; A case control study from central Norway. *BMC neurology* **8**, 1 (2008).

8 Abdelnour, C. *et al.* Alzheimer's disease cerebrospinal fluid biomarkers predict cognitive decline in Lewy body dementia. *Movement Disorders* **31**, 1203-1208 (2016).

9 Roquet, D., Sourty, M., Botzung, A., Armspach, J.-P. & Blanc, F. Brain perfusion in dementia with Lewy bodies and Alzheimer’s disease: an arterial spin labeling MRI study on prodromal and mild dementia stages. *Alzheimer's research & therapy* **8**, 29 (2016).

10 van der Flier, W. M. *et al.* Optimizing patient care and research: the Amsterdam Dementia Cohort. *Journal of Alzheimer's disease* **41**, 313-327 (2014).

11 Andersen, F. *et al.* Recruitment methods in Alzheimer's disease research: general practice versus population based screening by mail. *BMC medical research methodology* **10**, 1 (2010).

12 Idland, A.-V. *et al.* CSF neurofilament light levels predict hippocampal atrophy in cognitively healthy older adults. *Neurobiology of Aging* **49**, 138-144 (2017).

13 Pihlstrøm, L. *et al.* Supportive evidence for 11 loci from genome-wide association studies in Parkinson's disease. *Neurobiology of aging* **34**, 1708. e1707-1708. e1713 (2013).

14 Fladby, T. *et al.* Detecting at-risk Alzheimer’s disease cases. *Journal of Alzheimer's Disease* **60**, 97-105 (2017).

15 Morenas-Rodríguez, E. *et al.* Progranulin protein levels in cerebrospinal fluid in primary neurodegenerative dementias. *Journal of Alzheimer's Disease* **50**, 539-546 (2015).

16 Boada, M. *et al.* Design of a comprehensive Alzheimer’s disease clinic and research center in Spain to meet critical patient and family needs. *Alzheimer's & Dementia* **10**, 409-415 (2014).

17 Engedal, K. *et al.* Quantitative EEG applying the statistical recognition pattern method: a useful tool in dementia diagnostic workup. *Dementia and geriatric cognitive disorders* **40**, 1-12 (2015).

18 Jonsson, T. *et al.* Variant of TREM2 associated with the risk of Alzheimer's disease. *N Engl J Med* **368**, 107-116, doi:10.1056/NEJMoa1211103 (2013).

19 Organization, W. H. *The ICD-10 classification of mental and behavioural disorders: clinical descriptions and diagnostic guidelines*. Vol. 1 (World Health Organization, 1992).

20 American Psychiatric Association, A. & Association, A. P. Diagnostic and statistical manual of mental disorders. (1994).

21 McKeith, I. G. *et al.* Diagnosis and management of dementia with Lewy bodies: third report of the DLB Consortium. *Neurology* **65**, 1863-1872, doi:10.1212/01.wnl.0000187889.17253.b1 (2005).

22 McKeith, I. G. *et al.* Consensus guidelines for the clinical and pathologic diagnosis of dementia with Lewy bodies (DLB): report of the consortium on DLB international workshop. *Neurology* **47**, 1113-1124 (1996).

23 Van Steenoven, I. *et al.* Cerebrospinal Fluid Alzheimer’s Disease Biomarkers Across the Spectrum of Lewy Body Diseases: Results from a Large Multicenter Cohort. *Journal of Alzheimer's Disease*, 1-9 (2016).

24 Skogseth, R. E. *et al.* Accuracy of Clinical Diagnosis of Dementia with Lewy Bodies versus Neuropathology. *Journal of Alzheimer's Disease*, 1-14 (2017).

25 Watanabe, K., Taskesen, E., van Bochoven, A. & Posthuma, D. Functional mapping and annotation of genetic associations with FUMA. *Nat Commun* **8**, 1826, doi:10.1038/s41467-017-01261-5 (2017).

26 de Leeuw, C. A., Mooij, J. M., Heskes, T. & Posthuma, D. MAGMA: generalized gene-set analysis of GWAS data. *PLoS Comput Biol* **11**, e1004219, doi:10.1371/journal.pcbi.1004219 (2015).
